# Impact of Health Education on Soil-Transmitted Helminth Infections in Schoolchildren of the Peruvian Amazon: A Cluster-Randomized Controlled Trial

**DOI:** 10.1371/journal.pntd.0002397

**Published:** 2013-09-12

**Authors:** Theresa W. Gyorkos, Mathieu Maheu-Giroux, Brittany Blouin, Martin Casapia

**Affiliations:** 1 Division of Clinical Epidemiology, Research Institute of the McGill University Health Centre, Montreal, Quebec, Canada; 2 Department of Epidemiology, Biostatistics and Occupational Health, McGill University, Montreal, Quebec, Canada; 3 Department of Global Health and Population, Harvard School of Public Health, Boston, Massachusetts, United States of America; 4 Asociación Civil Selva Amazónica, Iquitos, Perú; University of Kelaniya, Sri Lanka

## Abstract

**Background:**

To control soil-transmitted helminth (STH) infections, the World Health Organization recommends school-based deworming programs with a health hygiene education component. The effect of such health hygiene interventions, however, has not been adequately studied. The objective of the present study was to determine the effectiveness of a health hygiene education intervention on the occurrence of STH re-infection four months post-de-worming.

**Methodology/Principal Findings:**

An open-label pair-matched cluster-randomized trial was conducted in Grade 5 schoolchildren of 18 primary schools (9 intervention and 9 control) in the Peruvian Amazon. Baseline assessment included interview with a pre-tested questionnaire and collection of single stool specimens that were examined using the single Kato-Katz thick smear. All schoolchildren were then treated with single-dose albendazole (400 mg). Schoolchildren in intervention schools then received 1) an initial one hour in-class activity on health hygiene and sanitation and 30-minute refresher activities every two weeks over four months; and 2) a half-day workshop for teachers and principals, while children in control schools did not. Four months later, STH infection was re-assessed in all schools by laboratory technologists blinded to intervention status. From April 21–October 20, 2010, a total of 1,089 schoolchildren (518 and 571 from intervention and control schools, respectively) participated in this study. Intervention children scored significantly higher on all aspects of a test of STH-related knowledge compared with control children (aOR = 18·4; 95% CI: 12·7 to 26·6). The intensity of *Ascaris lumbricoides* infection at follow-up was statistically significantly lower (by 58%) in children in intervention schools compared with children in control schools (aIRR = 0·42; 95% CI = 0·21 to 0·85). No significant changes in hookworm or *Trichuris trichiura* intensity were observed.

**Conclusions/Significance:**

A school-based health hygiene education intervention was effective in increasing STH knowledge and in reducing *Ascaris lumbricoides* infection. The benefits of school-based periodic deworming programs are likely to be enhanced when a sustained health hygiene education intervention is integrated into school curricula.

## Author Summary

The World Health Organization (WHO) recommends including a health hygiene education component into school-based deworming programs to reduce intestinal worm re-infection in treated children; however, the effect of these types of educational interventions has not been adequately studied. In this study, we investigated the effect of a health hygiene education intervention within a deworming program targeting Grade 5 schoolchildren in Bélen, Peru, a highly worm-endemic area. Following baseline assessment, all children in 18 primary schools received deworming. Subsequently, nine schools were randomly assigned to receive a health hygiene educational intervention and nine were randomly assigned to not receive the educational intervention. Four months later, children from schools that received the educational intervention were found to be more knowledgeable about the transmission and prevention of intestinal worm infections and, although there was no observed effect on whipworms or hookworms, children were also significantly less likely to be infected with roundworms. These results support the WHO recommendation for the inclusion of health hygiene education into school-based deworming programs. The beneficial effects of deworming are likely to be enhanced when appropriate health hygiene education is integrated into the school curricula.

## Introduction

Globally, soil-transmitted helminth (STH) infections (*Ascaris lumbricoides*, *Trichuris trichiura*, and hookworm) constitute one of the most important neglected tropical disease clusters of our time [1]. They affect over two billion people and cause significant morbidity and disability [2]. School-age children are considered the highest risk group as STH prevalence and intensity peak in the 5–14 year age group [3]. STHs are the leading cause of physical and intellectual growth and development delays and impairment of children in endemic areas [4], [5]. The World Health Organization (WHO), the United Nations Children's Fund (UNICEF), and the World Bank, among others, recommend deworming programs targeted to school-age children as the most cost-effective means to combat STH burden of disease [6]. In its listing of the most cost-effective investments targeting the top ten global challenges, the Copenhagen Consensus ranked deworming of schoolchildren as fourth among 16 interventions [7]. By treating the highest risk group, environmental contamination is reduced, and consequently, infection in the wider community decreases [8]. School-based deworming programs have been shown to contribute towards achieving several of the Millennium Development Goals (MDG) [9].

Despite effective treatment options for STH infections, re-infection following treatment alone is inevitable [10]. One strategy that has been identified to reduce re-infection following deworming treatment is health education, focussing on teaching hygienic and sanitary behaviours [10]–[12]. The inclusion of a health education strategy into school-based deworming programs is recommended [13], [14], based on the rationale that exposure to STH infections would be reduced and re-infection delayed through improved knowledge and behavioural changes [11], [15]. Health education strategies have been found to reduce the cost of deworming, and increase the level of overall health knowledge and acceptability of deworming interventions within the community [16]–[18]. Previous research investigating the knowledge, attitudes and practices of populations living in STH-endemic areas has revealed sub-optimal knowledge regarding STH infections; and, behaviours that could prevent STH infections are not being practiced [19], [20]. These results indicate that increased attention and research should be placed on health hygiene education integrated into deworming programs – to maximize the potential benefits.

Only five studies have previously investigated the efficacy or effectiveness of a health education intervention on STH prevalence or re-infection rates, with one study showing the benefits of such intervention on prevalence [21]–[25]. They, however, do not provide an adequate appreciation of the impact of a health education intervention because of methodological limitations (e.g. lack of randomization, no control group, no disaggregation of effect, or underpowered analysis).

The aim of the present study therefore was to investigate the effectiveness of a health education intervention targeted to schoolchildren on: 1) STH re-infection (the primary outcome); 2) general knowledge regarding STH infection and 3) STH-related behavioural change, at the individual level.

## Methods

### Ethics approval

This study was approved by the Research Ethics Board of the Research Institute of the McGill University Health Centre in Montréal, Canada and the Comité Institucional de Bioética of the Asociación Civil Impacta in Lima, Peru. This trial is reported in accordance with the CONSORT guidelines for cluster-randomized trials [26]. The trial is registered with *clinicaltrials.gov* (Registration number: NCT01085799). Written informed consent was obtained from each child's parent or guardian and written child assent was obtained from each child.

### Study area and participants

The study was conducted in Belén in the Peruvian Amazon, between April 21, 2010 and October 20, 2010. Belén is a peri-urban resource-poor community situated on the banks of the Itaya River. Due seasonal flooding, houses located in the low-lying areas of Belén (i.e. Belén Bajo) are constructed on wooden stilts or on floating platforms. Most inhabitants do not have access to reliable potable water for drinking nor adequate sanitation systems. Previous surveys have shown that STH prevalence is high in this community and particularly in the seasonally flooded area compared with the higher area (ie. Belén Alto) [27]–[29].

All primary schools in Belén were eligible for inclusion in the study. Inclusion criteria for schools were an enrollment of at least ten boys and ten girls in Grade 5. Grade 5 children were selected as the study population because of their mean age (approximately 10 years). This age group, more than any other across the lifespan, has been shown to have peak STH prevalence and intensity and, therefore, is the most representative age group to document the effect of control activities. Inclusion criteria for the schoolchildren were: i) enrolled in Grade 5; ii) parental informed consent; and iii) child assent. After school participation was confirmed with school principals, information sessions were organized at each school with the parents of all Grade 5 children. The study was explained and parental consent was requested. Child assent was requested from each child whose parent had provided informed consent prior to the baseline assessment.

### Study design and data collection

An open-label pair-matched cluster-randomized controlled trial study design was used. Baseline (April 21–June 16, 2010) and follow-up (at four months post-baseline: August 20–October 20, 2010) assessments were completed using the same study instruments. In brief, at baseline and at follow-up, a pre-tested and validated interviewer-administered questionnaire was used to collect demographic data, information on potential STH risk factors, and knowledge of STH transmission. Single stool specimens from participating children were collected and single smears analysed for STH infection and intensity using the Kato-Katz technique [13]. A total of three visits were made to each school at baseline (and four visits at follow-up) in order to give children multiple opportunities to participate if they were absent during previous visits. Research personnel assisted the children in obtaining the stool specimens and then transporting them to the laboratory where they were examined within 24 hours. Once slides were prepared (according to the Kato-Katz method), they were examined within 40 minutes. Quality control procedures were performed on 25% of all slides. Laboratory supervisors re-read these slides and discussed any discrepancies with laboratory technicians.

Following baseline assessment, all Grade 5 children were given a 400 mg chewable albendazole tablet (MicroLabs Ltd) by study personnel and each child was monitored to ensure that the tablet was chewed and swallowed. The tablet was administered the same day to children who had provided a stool specimen and on the third and final visit to children who had not been able to produce a stool specimen during any of the three visits. Efficacy of the albendazole treatment was assessed in a random sample of 385 children infected with at least one of the STH species two weeks following baseline visits [30].

A school questionnaire was administered to the school principal to collect information on the availability of gender-segregated bathroom facilities, water, soap, and on the level of bathroom cleanliness, in addition to information on school composition and organization.

### Randomization and masking

The unit of randomization was the school. To ensure a balanced proportion of children in each group and comparability between intervention and control schools with regard to expected baseline STH prevalence, schools were matched on Grade 5 student enrolment and geographical zone (Belén Bajo vs. Belén Alto). Within each pair, one school was randomly allocated to deworming and health education (intervention schools) and the other to deworming alone (control schools). The allocation sequence was generated automatically using a custom function that allocated schools using a random number generator with a binomial distribution in R statistical software (The R Project for Statistical Computing, http://www.r-project.org/). The randomization was executed by an independent statistician blinded to school identity. The laboratory technologists (primary outcome assessors) were blinded to intervention status.

### Health education intervention

The health education intervention was administered following the third baseline visit at each intervention school. It consisted of two components. First, in each Grade 5 classroom, a one-hour classroom activity was led by a member of the research team to describe STH acquisition, transmission and prevention. During this activity, a 32-page booklet (in Spanish) was given to each student and teacher. This booklet was inspired by the *Urbani School Health Kit* [31] and the *Escuelas Promotoras de Salud* strategy developed by the Peruvian Ministry of Health [32]. Second, a half-day workshop was organized for teachers and school principals with the goal of promoting an integrated health curriculum. These workshops were held on Saturdays following the baseline deworming. Teachers' resource booklets were provided and discussed. They were adapted from the *Urbani School Kit* and focussed on how to develop creative ways to help children improve their personal hygiene and understand the importance of preventing STH infection [33].

Intervention schools were visited every two weeks between baseline and follow-up study visits during which students were reminded of what they had been taught during the class activity and were encouraged to practice what they had learned. Posters highlighting key health messages were distributed and displayed in strategic locations around the school. All education materials were pre-tested in similar schools of a neighbouring district.

At the end of the study, control schools were offered the health education intervention and children in all grades in both intervention and control schools were dewormed.

### Statistical analysis

Due to the clustering by schools, a design effect was calculated from the intraclass correlation coefficient (ICC) to account for within-school clustering. As no reported ICCs of within-school clustering of STH re-infection were found in the literature, previous STH data from Belén [27] from 1,074 Grade 5 students were used to calculate the within-school ICC of STH prevalence. The obtained ICC was 0·028, corresponding to a design effect of 2·77. The sample size calculation was based on the formula for logistic regression with a single binary covariate (i.e. the health education intervention) and the Wald test was used as the basis for computation. Assuming that 50% of the children would be exposed to the intervention, that the re-infection rate in the control group would be 49·5% [34], that there would be an expected 5% loss to follow-up at 4 months, and accounting for a design effect of 2·77, a sample size of 1,101 would have good power (80·3%) to detect a moderate effect (i.e. OR = 0·57) and would have an excellent power (93·4%) to detect a strong effect (i.e. OR = 0·50).

A proxy for socio-economic status (SES) was constructed based on a Principal Component Analysis (PCA) of student-reported family asset ownership [35]. The asset variables selected for this analysis included: house material, use of gas for cooking, presence of electricity in the home, radio ownership, television ownership and number of persons living in the home. The first PCA axis explained 36% of the variance in asset ownership. This asset-based index was then categorized into quartiles.

Descriptive statistics, including population means, standard deviations and proportions (as appropriate) are presented to describe the study population. Both arithmetic and geometric means of STH intensity (ie. eggs per gram of stool) are presented to increase comparability with other studies.

Univariate results are presented as the difference in means of outcome variables between intervention and control groups with 95% confidence intervals. The 95% confidence intervals of the difference between two geometric means were obtained using 9,999 bootstrap replicates.

Clustering of standard errors at school level and the matched-pair design were taken into account through the use of multivariable random effects regression models and the inclusion of the pairs as fixed effects in all regression models, respectively. Random effects ordinal regression (also known as cumulative link mixed model) was used to determine the effect of the intervention on STH-related knowledge; random effects logistic regression, on behavioural outcomes and STH prevalence; and random effects negative binomial regression, on STH intensity. In order to improve the efficiency of the effect size estimates for the intervention, all multivariable models were adjusted for all known and measured confounders that were judged to have sufficient variation, few missing data, and small measurement error. These included: age, sex, SES status, presence of running water in the home, baseline values of outcome measures (e.g. baseline STH values, baseline knowledge values, etc.), time of year of baseline visit and length of follow-up. Effect modification of the intervention by sex was also explored. Potential contamination between intervention and control schools (i.e. spillover effects) was investigated by regressing, among control schools, categorized distance to the closest intervention schools for each outcome. Observations with missing variables (i.e. individual-level information) were excluded from the analyses (n = 2). Statistical significance was assessed at p<0·05. Intracluster correlation coefficients (ICCs) for STH infections at follow-up (in the control schools) were calculated using a generalized mixed model and model linearization was used to calculate variance components. Non-parametric confidence intervals for the ICC were obtained using 9,999 bootstrap replicates.

All analyses were performed using the R statistical software, version 2·15·1. The ‘lme4’ library was used to fit the logistic random effect models; the ‘ordinal’ library was used to fit the ordinal random effects model; the ‘glmmAMDB’ library was used to fit the random effects negative binomial models, and the ‘aod’ and ‘boot’ libraries were used to calculate the ICCs.

## Results

**Figure 1:**
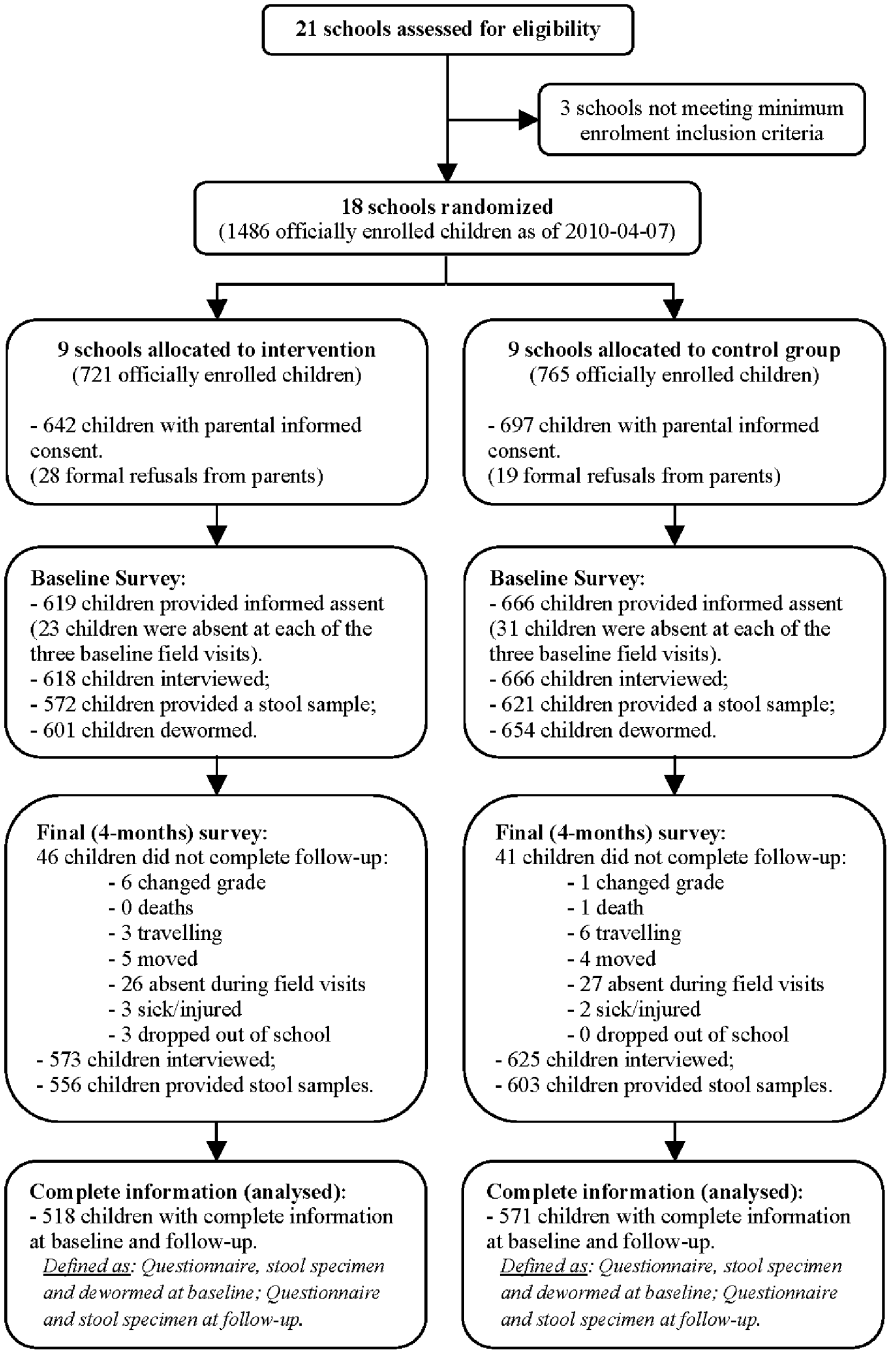
Trial profile.

Of the 21 primary schools in Belén, 18 were eligible based on minimum enrolment criteria and were randomized to either the intervention (N = 9) or control group (N = 9) (Figure 1). Of the 1,486 officially enrolled children, informed consent was obtained from 1,339 parents (90·1%) and child assent was obtained from 1,286 students (86·5%). Complete data (including baseline questionnaire, baseline stool specimen, dewormed at baseline, follow-up questionnaire and follow-up stool specimen) were obtained for 1,089 children, or 84·7% of those who assented (518 in the intervention group and 571 in the control group). Baseline information, collected before the initial deworming, for intervention and controls groups for participating children and schools, is presented in Table 1.

**Table 1:** Baseline characteristics of Grade 5 students who completed baseline and follow-up assessments and were dewormed following baseline assessment (n = 1,089), and of participating schools (n = 18), by intervention status, in Belen, Peru, April–June 2010.

Variables	Intervention	Control
**Individualcharacteristics**	**(n = 518)**	**(n = 571)**
Sex (% female)	46·0	50·8
Age [mean (sd)]	10·2 (1·1)	10·4 (1·1)
Weight (kg) [mean (sd)]	31·8 (6·3)	32·0 (6·8)
Height (cm) [mean (sd)]	134·3 (6·9)	134·4 (7·4)
House material (% Brick)	40·7	30·8
Running water in home (%)	74·3	75·0
Have been dewormed in past (%)	64·8	63·6
Number of people in household [mean (sd)]	7·0 (2·9)	7·3 (3·2)
Number of <5 year olds in household [mean (sd)]	0·8 (0·9)	0·9 (1·0)
*Socio-Economic Status*		
Lowest Quartile (%)	30·2	22·6
Second Quartile (%)	24·8	24·5
Third Quartile (%)	21·3	26·1
Highest Quartile (%)	23·8	26·8
*Ascaris lumbricoides*		
Prevalence (%)	49·2	55·5
Intensity (EPG)* [mean (sd)]	8456 (17744)	9968 (22020)
*Trichuris trichiura*		
Prevalence (%)	59·1	64·8
Intensity (EPG)* [mean (sd)]	1045 (2841)	832 (2344)
*Hookworm*		
Prevalence (%)	12·0	12·6
Intensity (EPG)* [mean (sd)]	43 (194)	52 (417)
*Any STH infection*		
Prevalence (%)	72·2	78·1
**Schoolcharacteristics**	**(N = 9)**	**(N = 9)**
Zone (% in flooding zone)	44·4	44·4
Number of levels in school [mean (sd)]	1·44 (0·88)	1·89 (1·05)
Number of students (Grade 1–6) [mean (sd)]	516·7 (384·8)	551·3 (421·8)
Water available in school (%)	88·9	88·9
Water is treated (%)	44·4	11·1
Bathroom present in school (%)	88·9	100
General hygiene score of bathrooms (score out of 8) [mean (sd)]	2·25 (1·98)	2·2 (0·97)
Soap available in school (%)	22·2	22·2

* *EPG: eggs per gram; arithmetic mean*.

The efficacy of albendazole results are discussed in full elsewhere [30]. Briefly, albendazole was highly efficacious against *Ascaris lumbricoides* infection (mean egg reduction rates (ERR) = 98.8%; 95% CI: 92.1, 99.9) and hookworm infection (ERR = 86.3%; 95% CI: 71.6, 93.0); and it was moderately efficacious against *Trichuris trichiura* infection (ERR = 42.2%; 95% CI: 25.6, 53.5).

At follow-up, children who had received the health hygiene education intervention scored significantly higher on all aspects of a test of STH-related knowledge compared children who had not received the intervention (Table 2). Children in the intervention group scored, on average, 5·3 points higher, out of a possible score of 12 (i.e. 44% higher) than children in the control group. This result remained significant in the multivariable analysis (aOR = 18·4; 95% CI: 12·7 to 26·6). At follow-up, the odds of having a one point increase in score was, on average, 18 times higher in the intervention schools compared with the control schools.

**Table 2:** STH knowledge scores^a^ at baseline and follow-up (4 months post-deworming), by intervention group, Grade 5 schoolchildren in Belen, Peru, 2010.

Variables	Intervention (n = 518)	Control (n = 571)	Mean difference between intervention and control groups
**Baseline**	*Mean (SD)*	*Mean (SD)*	*Mean (95% CI)*
Knows how worms are acquired^b^	0·147 (0·52)	0·142 (0·57)	0·005 (−0·060, 0·069)
Knows why worms are bad for your health^b^	0·151 (0·52)	0·130 (0·51)	0·021 (−0·041, 0·083)
Knows how to prevent worms^b^	0·405 (0·63)	0·504 (0·67)	−0·099 (−0·176, −0·022)*
Total Score^c^	0·703 (1·20)	0·776 (1·21)	−0·073 (−0·703, 0·776)
**Follow-Up**			
Knows how worms are acquired^b^	2·651 (1·40)	0·434 (0·91)	2·217 (2·074, 2·358)*
Knows why worms are bad for your health^b^	1·591 (1·43)	0·349 (0·79)	1·242 (1·103, 1·382)*
Knows how to prevent worms^b^	2·514 (1·40)	0·695 (0·85)	1·819 (1·679, 1·958)*
Total Score^c^	6·755 (3·51)	1·478 (2·04)	5·277 (4·930, 5·623)*

* *Statistically significant difference between intervention and control groups*.

a *Scores were generated by asking children three open-ended questions related to STH infection and prevention*.

b *Score out of 4*.

c *Score out of 12*.

In univariate analyses, the increased knowledge in children in the intervention group translated into behavioural change with regard to treating water before drinking it and bathing in the river (Table 3). At follow-up, children who had received the intervention were less likely to drink water directly (without treating it) and were less likely to bathe in the river compared with children who had not received the intervention. In multivariable analysis, children in the intervention schools had a 0·58 decreased odds of reporting consuming water directly compared with children in the control schools (aOR = 0·58; 95% CI: 0·44 to 0·76). Children in the intervention schools had a 0·72 decreased odds of reporting bathing in the river at follow-up compared with children in the control schools; however, this result did not reach statistical significance (aOR = 0·72; 95% CI: 0·48 to 1·06). No other statistically significant differences in STH-related behaviours between intervention and control groups were observed.

**Table 3:** Behavioural factors at follow-up (4 months post-deworming), by intervention group, Grade 5 schoolchildren in Belen, Peru, 2010.

Behaviours	Intervention	Control	Difference between proportions^a^
	(n = 518)	(n = 571)	[difference (95% CI)]
Has dirty nails [#, (%)]	128 (24·7)	128 (22·4)	2·3 (−2·9, 7·5)
Drinks water directly [#, (%)]	253 (48·8)	357 (62·5)	−13·7 (−19·7, −7·6)*
Bathes in Itaya river [#, (%)]	Always: 47 (9·1)	Always: 50 (8·8)	0·3 (−3·2, 3·9)
	Sometimes: 120 (23·2)	Sometimes: 190 (33·3)	−10·1 (−15·6, −4·6)*
	Never: 351 (67·8)	Never: 331 (58·0)	9·8 (3·9, 15·7)*
Defecates in open air [#, (%)]	Always: 6 (1·2)	Always: 3 (0·53)	0·7 (−0·6, 1·9)
	Sometimes: 47 (9·1)	Sometimes: 48 (8·4)	0·7 (−2·9, 4·2)
	Never: 465 (89·8)	Never: 520 (91·1)	−1·3 (−5·0, 2·4)
Uses toilet paper when defecates	Always: 458 (88·4)	Always: 502 (87·9)	0·5 (−3·5, 4·5)
[#, (%)]	Sometimes: 57 (11·0)	Sometimes: 69 (12·1)	−1·1 (−5·1, 2·9)
	Never: 3 (0·6)	Never: 0 (0)	0·6 (−0·3, 1·4)
Washes hands after going to	Always: 413 (79·7)	Always: 440 (77·1)	2·6 (−2·4, 7·7)
bathroom [#, (%)]	Sometimes: 100 (19·3)	Sometimes: 127 (22·2)	−2·9 (−7·9, 2·1)
	Never: 5 (1·0)	Never: 4 (0·7)	0·3 (−1·0, 1·5)
Uses soap when washing hands	Always: 358 (69·1)	Always: 394 (69·0)	0·1 (−5·5, 5·7)
after going to bathroom [#, (%)]	Sometimes: 137 (26·4)	Sometimes: 154 (27·0)	−0·6 (−6·0, 4·9)
	Never: 23 (4·4)	Never: 23 (4·0)	0·4 (−2·2, 3·0)
Washes hands before eating	Always: 402 (77·6)	Always: 449 (78·6)	−1·0 (−6·1, 4·1)
[#, (%)]	Sometimes: 111 (21·4)	Sometimes: 120 (21·0)	0·4 (−4·6, 5·5)
	Never: 5 (1·0)	Never: 2 (0·4)	0·6 (−0·5, 1·8)
Uses soap when washing hands	Always: 355 (68·5)	Always: 397 (69·5)	−1·0 (−6·7, 4·7)
before eating [#, (%)]	Sometimes: 120 (23·2)	Sometimes: 133 (23·3)	0·0 (−5·3, 5·0)
	Never: 43 (8·3)	Never: 41 (7·2)	1·1 (−2·2, 4·5)
Washes fruit before eating [#, (%)]	Always: 418 (80·7)	Always: 460 (80·6)	0·1 (−4·7, 5·0)
	Sometimes: 89 (17·2)	Sometimes: 103 (18·0)	−0·8 (−5·6, 3·9)
	Never: 8 (1·5)	Never: 8 (1·4)	0·1 (−1·4, 1·7)
Walks barefoot [#, (%)]	Always: 48 (9·3)	Always: 52 (9·1)	0·2 (−3·4, 3·8)
	Sometimes: 299 (57·7)	Sometimes: 336 (58·8)	−1·1 (−7·2, 4·9)
	Never: 171 (33·0)	Never: 183 (32·0)	1·0 (−4·8, 6·7)
Wears shoes/sandals at home	508 (98·1)	566 (99·1)	−1·0 (−2·6, 0·5)
[#, (%)]			

a *Difference between intervention and control groups*.

* *Statistically significant difference between intervention and control groups*.

While univariate comparisons of STH infection between intervention and control schools did not show statistically significant differences (Table 4), in multivariable analyses, children in intervention schools had a statistically significant decrease in *Ascaris lumbricoides* intensity compared with children from control schools (aIRR = 0·42; 95% CI = 0·21 to 0·85) (Table 5). At follow-up, the intensity of *Ascaris lumbricoides* infection in children from intervention schools was 58% lower than children from control schools. There were no statistically significant differences observed between children of intervention and control schools in the intensities of either *Trichuris trichiura* or hookworm infection (Table 5), nor in the prevalences of any STH infection (Table 6). No statistically significant differences in the effect of the intervention on STH infection were observed between the sexes (data not shown). The regression analysis for spillover effects revealed that contamination between intervention and control schools had not occurred (data not shown). Intracluster correlation coefficients for each STH prevalence are shown in Table 7.

**Table 4:** STH data at follow-up (four months post-deworming) by intervention group, Grade 5 schoolchildren in Belen, Peru, 2010, univariate results.

STH Variable	Intervention	Control	Difference between means/proportions^a^
	(n = 518)	(n = 571)	[difference (95% CI)]
***Ascarislumbricoides***			
Prevalence (%)	31·9	36·4	−4·5 (−10·4, 1·2)
Intensity (AM*) [mean (sd)]	1392 (5927)	2147 (7206)	−755 (−27, 1536)
Intensity (GM †) [mean]	1182·3	1428·6	−246·3 (−730·6, 227·9)
FECR§) [mean (sd)]	7095 (18,088)	7790 (21,772)	−695 (−1678, 3067)
Intensity Levels [#, (%)]	Heavy: 2 (0·4)	Heavy: 4 (0·7)	−0·3 (−1·4, 0·7)
	Moderate: 32 (6·2)	Moderate: 53 (2·6)	3·6 (−6·4, 0·2)
	Low: 131 (25·3)	Low: 151 (26·4)	−1·1 (−6·5, 4·2)
	None: 353 (68·1)	None: 363 (63·6)	4·5 (−1·2, 10·4)
***Trichuristrichiura***			
Prevalence (%)	52·1	56·7	−4·6 (−10·7, 1·5)
Intensity (AM*) [mean (sd)]	450·6 (1659)	309·8 (760)	140·8 (−297·0, 15·4)
Intensity (GM†) [mean]	286·2	225·7	60·5 (4·21, 120·15)
FECR§ [mean (sd)]	577·0 (2,343)	538·8 (2,116)	38·2 (−304·6, 228·4)
Intensity Levels [#, (%)]	Heavy: 2 (0·4)	Heavy: 0 (0)	0·4 (−0·3, 1·1)
	Moderate: 56 (10·8)	Moderate: 54 (9·5)	1·3 (−2·4, 5·1)
	Low: 212 (40·9)	Low: 270 (47·3)	−6·4 (−12·4, −0·2)
	None: 248 (47·9)	None: 247 (43·3)	4·6 (−1·5, 10·7)
**Hookworm**			
Prevalence (%)	6·6	4·9	1·7 (−1·3, 4·6)
Intensity (AM*) [mean (sd)]	11·2 (70)	7·9 (73)	3·3 (−11·8, 5·3)
Intensity (GM †) [mean]	107·5	81·9	25·6 (−21·3, 71·5)
FECR§) [mean (sd)]	32·3 (187)	43·9 (420)	25·6 (−26·5, 49·8)
Intensity Levels [#, (%)]	Heavy: 0 (0)	Heavy: 0 (0)	0 (0, 0)
	Moderate: 0 (0)	Moderate: 0 (0)	0 (0, 0)
	Low: 34 (6·6)	Low: 28 (4·9)	1·7 (−1·3, 4·6)
	None: 484 (93·4)	None: 543 (95·1)	−1·7 (−4·6, 1·3)
**Any STH infection**			
Prevalence (%)	62·6	67·6	−5·0 (−10·9, 0·8)

a *Mean difference between intervention and control groups*.

* *AM: Arithmetic Mean*.

† *GM: Geometric Mean calculated with infected students only*.

§ *FECR: Fecal Egg Count Reduction: the mean of individual differences between baseline EPG (eggs per gram) and follow-up EPG*.

**Table 5:** Effect of the health hygiene intervention on STH intensity (EPG†) at follow-up (four months post-deworming) in multivariable random effects negative binomial regression models.

Outcome variable	Unadjusted	Adjusted
	IRR (95% CI)	aIRR^a^ 95% CI
*Ascaris lumbricoides* intensity (EPG†)	0·52 (0·27–1·01)	0·42 (0·21 to 0·85)*
*Trichuris trichiura* intensity (EPG†)	1·56 (0·98–2·48)	1·14 (0·78 to 1·67)
Hookworm intensity (EPG†)	1·57 (0·28–8·81)	0·11 (0·01 to 1·49)

a *Incidence rate ratios (IRR) adjusted for sex, SES status, age, running water in the home, time of year of baseline visit, length of follow-up, baseline parasitological intensity (epg), random effects at the school level, and fixed effect for the matched-pairs*.

† *EPG: eggs per gram*.

* *Statistically significant effect measure*.

**Table 6:** Effect of the health hygiene intervention on STH prevalence at follow-up (four months post-deworming) in multivariable random effects logistic regression models.

Outcome variable	Unadjusted	Adjusted
	OR (95% CI)	aOR* 95% CI
*Ascaris lumbricoides* prevalence	1·00 (0·61–1·34)	0·88 (0·57 to 1·34)
*Trichuris trichiura* prevalence	1·05 (0·60–1·83)	0·88 (0·62 to 1·25)
Hookworm prevalence	1·66 (0·95–2·89)	1·13 (0·51 to 2·50)
Any STH prevalence	1·10 (0·57–2·14)	1·00 (0·58 to 1·72)

* *Odds ratios adjusted for sex, SES status, age, running water in the home, time of year of baseline visit, length of follow-up, baseline parasitological prevalence, random effects at the school level, and fixed effect for the matched-pairs*.

**Table 7:** Intracluster correlation coefficients (ICCs) for STH re-infection prevalences, four months post-deworming (N = 571, from the 9 control schools).

STH	ICC	95% CI
*Ascaris lumbricoides*	0·042	(0·009 to 0·085)
*Trichuris trichiura*	0·127	(0·062 to 0·231)
Hookworm	0·033	(0·000 to 0·067)
Any STH	0·121	(0·046 to 0·229)

## Discussion

This study examined the effect of a post-deworming hygiene education intervention on the level of STH-related knowledge, risk behaviours and the prevalence and intensity of STH re-infection using a pair-matched cluster-randomized controlled trial design. Compared with children from control schools, at follow-up (i.e. four months after deworming), children from intervention schools had higher scores on a test of STH-related knowledge, were less likely to bathe in the contaminated river, were more likely to treat/boil water before consumption, and had lower intensity levels of *Ascaris lumbricoides* infection. These results suggest that the health hygiene educational intervention was successful at improving Grade 5 children's knowledge regarding STHs and that this increase in knowledge translated to some improvements in health hygiene behaviours which led to a reduced intensity of *Ascaris lumbricoides* infection. No reduction was observed in either the intensity of *Trichuris trichiura* or hookworm infections. This was somewhat expected for *Trichuris trichiura* given that albendazole has been shown to have very low cure rates (i.e. approximately 28%, compared with 88% for *Ascaris lumbricoides* and 72% for hookworm) [36]. The relatively low prevalence of hookworm in our study population and the fact that the observed behaviour changes in the intervention schools were not found to affect its mode of transmission (i.e. contact with bare skin) could explain the lack of significant effect on intensity of hookworm infection. In addition, we only used one day's stool collection for outcome assessments, and, although this will yield very good accuracy for *Ascaris lumbricoides* and *Trichuris trichiura*, the moderate sensitivity of this technique for hookworm (i.e. 65.2%) could have biased our results [37].

Although this study documented reduced *Ascaris lumbricoides* intensity levels following the educational intervention, no statistically significant differences in prevalences were found. Intensity is, in fact, a more appropriate indicator of morbidity than STH prevalence and deworming programs are designed primarily to reduce population-level infection intensity levels rather than prevalence. It is inevitable that children will become re-infected following deworming (especially in the first few cycles of a deworming program); however, a decrease in intensity levels represents reduced morbidity and is indicative of important health benefits [38]. Behaviour change is a long-term outcome measure and a longer follow-up period, with continued education, would likely produce more marked changes in behaviour and subsequent decreases in STH infection (in both intensity and, eventually, prevalence).

The major strengths of this study include the large sample size, the randomized design, the high response and follow-up rates, and, appropriate statistical adjustment for clustering by schools, the matched-paired design, and potential confounders. One limitation of this study is related to the open-label design. Although difficult to assess, it may be possible that students' knowledge of their exposure status (i.e. whether they received the intervention or not) may have biased their self-reported responses to the behaviour questions, leading to potential measurement error. Another limitation is that we only had one follow-up period (i.e. four months) and we cannot conclude if knowledge of STH transmission and the protective effects on intensity of *Ascaris lumbricoides* intensity of infection can be maintained over longer time periods. Finally, STH-related behaviours were measured by self-report, which may have introduced measurement error, especially in the intervention group. Due to the large sample size, and feasibility issues, it was not possible to directly observe the behaviours. Self-report from children aged 8–11 years, however, has been found to be a reliable and valid measurement tool in the context of health-related questionnaires [39].

This study documents the effectiveness of a health hygiene educational intervention, integrated into a school-based deworming program. Our results support the WHO recommendation to include hygiene education into deworming programs. More research is needed to find better ways of translating improved knowledge into sustained behavioural changes. Social marketing at the community level and further involvement of the children's parents in the education intervention could potentially maximize behavioural changes and lead to greater reductions in the burden of STH infection in endemic areas.

## Supporting Information

Protocol S1 Detailed research protocol.
(DOCX)

Checklist S1 CONSORT checklist.
(DOC)
