# Supplementary material for: Impact of Health Education on Soil-Transmitted Helminth Infections in Schoolchildren of the Peruvian Amazon: A Cluster-Randomized Controlled Trial
Source: PLoS Negl Trop Dis. 2013 Sep 12;7(9):e2397. doi: 10.1371/journal.pntd.0002397 (PMC3772033; doi:10.1371/journal.pntd.0002397)
Supplement: Protocol S1 — Detailed research protocol. (DOCX) [file pntd.0002397.s001.docx]

Effectiveness of a post-deworming education intervention to reduce soil-transmitted helminth infections and absenteeism in grade 5 school-children in a community of extreme poverty, Peruvian Amazon

**Martín Casapía ^1^** – Project Director

**Mathieu Maheu-Giroux ^2^** – Research Coordinator (Canada)

**Theresa W. Gyorkos ^2, 3^** – Principal Investigator

1- Asociación Civil Selva Amazónica, Iquitos, Perú

2- Division of Clinical Epidemiology, McGill University Health Centre, Montreal, QC, Canada

3- Department of Epidemiology, Biostatistics, and Occupational Health of McGill University, Montreal, QC, Canada

December 2009

**Table of Contents**


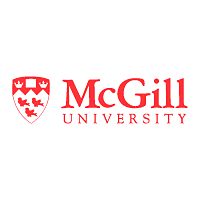

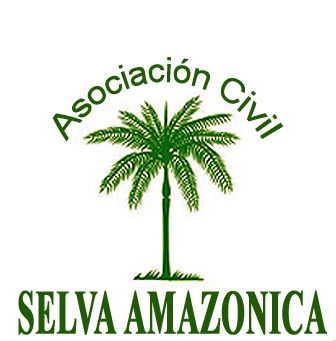

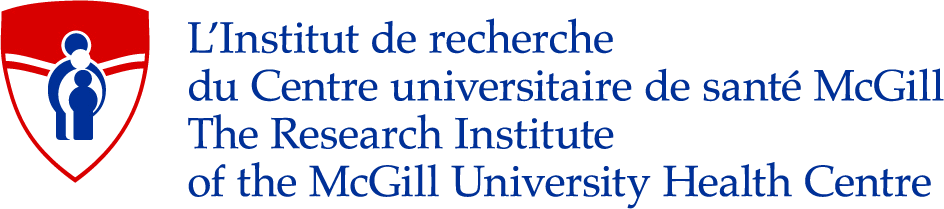


[**Executive summary** 2](#_Toc352673108)

[**Context of the present protocol submission** 2](#_Toc352673109)

[**Location of study conduct** 2](#_Toc352673110)

[**Study rationale** 2](#_Toc352673111)

[**Scientific background** 3](#_Toc352673112)

[**Objectives** 5](#_Toc352673113)

[**Primary** 5](#_Toc352673114)

[**Secondary** 5](#_Toc352673115)

[**Methods** 5](#_Toc352673116)

[**Study design** 5](#_Toc352673117)

[**Study population** 5](#_Toc352673118)

[*Inclusion criteria* 6](#_Toc352673119)

[*Exclusion criteria* 6](#_Toc352673120)

[**Health hygiene education intervention** 6](#_Toc352673121)

[**Measurements** 7](#_Toc352673122)

[**Sample size justification** 8](#_Toc352673123)

[**Planned statistical analyses** 9](#_Toc352673124)

[**Safety reporting** 9](#_Toc352673125)

[**Anticipated benefits** 10](#_Toc352673126)

[**Potential risks** 10](#_Toc352673127)

[**Privacy and confidentiality** 10](#_Toc352673128)

[**Details of trial team** 10](#_Toc352673129)

[**Ethical oversight** 11](#_Toc352673130)

[**Compensation** 11](#_Toc352673131)

[**Results dissemination** 11](#_Toc352673132)

[**Funding** 11](#_Toc352673133)

[**Chronogram of research activities** 12](#_Toc352673134)

[**References** 13](#_Toc352673135)

[**Annexes** 17](#_Toc352673136)

[**Annex 1:** Informed Consent Form - Parents (Spanish Version) 18](#_Toc352673137)

[**Annex 2:** Informed Consent Form - Parents (English Version) 21](#_Toc352673138)

[**Annex 3:** Informed Assent Form - Students (Spanish Version) 24](#_Toc352673139)

[**Annex 4:** Informed Assent Form – Students (English Version) 26](#_Toc352673140)

[**Annex 5:** Questionnaire (Spanish Version) 28](#_Toc352673141)

[**Annex 6:** Questionnaire (English Version) 32](#_Toc352673142)

**Executive summary**

Belén, a community of extreme poverty in Peru, has listed intestinal parasites such as soil-transmitted helminths (STH) (*Ascaris, Trichuris*, and hookworm) among their top-five health priorities during recent multidisciplinary participatory workshops [1]. Furthermore, a school survey conducted in 2006 in this community demonstrated a very high prevalence of STH infections, with 86% of Grade 5 students infected with one or more species of parasites [2]. To efficiently control this disease cluster, WHO, PAHO, and others recommend the inclusion of an education strategy in school-based deworming programs [3-6]. However, the effectiveness of such a strategy on the rate of STH re-infection and on education indicators, such as absenteeism, remains to be fully understood. The proposed research aims to evaluate the effectiveness of a post-deworming education intervention targeted to Grade 5 school children enrolled in Belén’s schools using a cluster-randomized trial design. Results will be used to inform school-based deworming programs in Peru and other similar endemic areas in Latin America and, indeed, around the world.

**Context of the present protocol submission**

The present submission details a 4-month project based entirely in Peru, which will be conducted within a larger 5-year research program (2006-2011) already funded by the CIHR (RI-MUHC account 6360). The 5-year project is an Interdisciplinary Capacity Enhancement (ICE) grant. As such, the ICE project included overall objectives only. The plan was to develop specific projects within the ICE grant in conjunction with local co-investigators and collaborators, and the local community. Ethics review was not required for the overall 5-year project. Instead, as projects would be developed, they would individually be submitted for appropriate ethics approval, both in Peru and in Canada. Ethics approval from Peruvian Health officials for the current project is underway. English and Spanish versions of the parents/tutor informed consent form, the students assent form and the questionnaire are attached in the Annex.

**Location of study conduct**

The Belén district of Iquitos is located on the banks of the Itaya/Amazon River in Peru. Due to the propensity of this area for seasonal flooding, the houses are constructed on wooden stilts or on floating platforms. Most inhabitants do not have access to reliable potable water for drinking or improved sanitation systems. Human waste contaminates the water directly from floating latrines or indirectly from land-based latrines, leading to a state of extensive fecal contamination.

**Study rationale**

Because of the very high prevalence of STH in Grade 5 school children in Belén, Peru, efficient and sustainable control methods are needed. There has been no systematic review on the proposed research topic. Few studies have investigated the effectiveness of a post-deworming education intervention on STH (*Ascaris, Trichuris*, and/or hookworm) prevalence or re-infection rates, or on absenteeism, and only five studies have previously examined school-based education interventions. These studies yielded mixed evidence and results are difficult to interpret because of methodological limitations. Therefore, in this current state of scientific equipoise, our proposed cluster-randomized trial is needed to inform both policy and practice related to school-based deworming programs in Peru and in other endemic countries.

**Scientific background**

Globally, STH (*Ascaris, Trichuris*, and hookworm) infections are one of the most important neglected disease clusters worldwide as they affect over two billion people and they contribute significant morbidity and disability, especially in the high risk group of school-age children [7]. STH are the leading cause of physical and intellectual growth and development delays and impairment of children in endemic areas [8-10]. Furthermore, it is in school-age children that peak prevalences and peak intensities of STH infections occur [11]. The World Health Organization (WHO), UNICEF, and the World Bank, among others, recommend establishing school-based deworming programs as the most cost-effective means to combat the adverse health outcomes of these parasitic diseases. The rationale for these school-based interventions is to reduce the highest STH-attributable burden of disease (by treating the highest risk group of school-age children). By doing so, this intervention also reduces environmental contamination, and consequently, infection in the wider community [12]. Further, school-based deworming programs have been shown to be “a crucial, and neglected, step towards improving public health and to reaching several of the Millennium Development Goals” (MDG) [13, 14], which call for concerted improvements in poverty reduction, nutritional status, education, gender equality, and environmental degradation. For example, treatment with deworming drugs (eg. albendazole) led to a 25% reduction in primary school absenteeism in Kenya [15], prevented 82% of stunting and was associated with a 35% weight gain among children in Indian slums [16]. Despite their usefulness, school-based deworming programs have several constraints: many endemic communities don’t have them (only 64 of 130 endemic countries reported any school-based deworming activity at all in 2006); positive effects are temporary (in the absence of any behavioural or environmental change, due to re-infection) [17], thereby requiring repeated treatment; and deworming programs incur costs, even if the drug itself is free of charge or of low cost [3, 18].

The inclusion of a health education strategy in school-based deworming programs is therefore recommended as a way to improve their effectiveness [3-6, 19]. The specific objectives of the health education strategy are to prevent re-infection and improve school effects, like absenteeism. STH infections being acquired through the fecal-oral transmission route (*Ascaris* and *Trichuris*) or contact with contaminated soil (hookworm), the approach of health education programs is to promote behavioural changes that would reduce exposure and risk of contamination. Other advantages of these education programs is that they can reduce the cost of deworming, increase the level of overall health knowledge within the community, reduce morbidity attributable to STH infections and promote optimal productivity as adults [6, 20, 21].

A literature review on the efficacy or effectiveness of school-based health education interventions on the prevalence or infection rates of STH infections retrieved only five studies, none of them from Latin America. An annotated summary of these studies demonstrates a state of scientific equipoise regarding our proposed research question (Table 1). Furthermore, evidence from these studies is difficult to interpret and to generalize because: some did not have appropriate comparison groups; the health education intervention was sometimes implemented together with environmental sanitation programs or other interventions; there was often no statistical adjustment for confounders nor any correction for within-school clustering of students; some had low sample sizes, targeted different age groups, had variable follow-up periods and were often only descriptive in nature. The cumulative evidence is therefore equivocal, not only because some studies showed a negative effect, an absence of effect, or a positive effect of the health education intervention on STH prevalence or re-infection rates, but also because the methodological basis for this evidence is less than rigorous.

Table 1: Annotated review of studies conducted on the efficacy or effectiveness of health education intervention on STH prevalence or re-infection rates

| **Location (Year)** | **Study Design** | **# schools** | **# children** | **Limitations** | **Results** | **Ref.** |
| --- | --- | --- | --- | --- | --- | --- |
| China  (1997) | Observational | 6 schools  (4 received the intervention) | 6,188 | - No adjustment for school clustering  - No measure of effect reported  - Intervention consisted of a health intervention plus environmental sanitation | Schools with the environmental sanitation and health intervention had lower re-infection rates. | [22] |
| Indonesia  (2005) | Observational | 45 schools  (5 received the intervention) | 3,463 | - Descriptive study  - No statistical analysis  - No adjustment for potential individual confounders | Schools receiving the health education intervention had lower prevalence at 7 months | [23] |
| Indonesia  (1998) | Factorial RCT | 5 schools  (4 treated and 1 control) | 336 | - Individual confounders not taken into account  - Low sample size | No effect differences were found between the ‘deworming group’ and the ‘deworming plus education’ group | [24] |
| Seychelles  (1994) | Observational | 23 schools  (all received the intervention) | 1,244 | - No comparison group  - No statistical analysis  - Intervention consisted of health education, environmental sanitation, and deworming | Deworming, environmental sanitation, and health education reduces the prevalence of STH | [25] |
| Thailand  (2004) | Observational | 4 schools  (all received the intervention) | 428 | - No comparison group  - Low sample size | Prevalence of STH was higher at the end of the health education intervention compared to baseline | [26] |

**Objectives**

**Primary**

The primary objective is to determine the effectiveness of a school-based health hygiene education intervention to reduce re-infection by STH after a routine deworming intervention.

**Secondary**

The following secondary research objectives will also be addressed: i) to determine intervention effects on absenteeism rates; ii) to assess gender-specific determinants of STH infection, re-infection and absenteeism rates; iii) to measure STH species-specific cure rates and egg reduction rates; and iv) to examine behavioural and social determinants of STH infection, re-infection and absenteeism rates.

**Methods**

**Study design**

A prospective cluster-randomized controlled trial design will be used (Figure 1). Schools will be randomly allocated to one of the two arms using a stratified randomization technique (by school size). Because the total number of students enrolled in Grade 5 varies from 20 to more than 200 per school, the stratified design will ensure a balanced proportion of children in each of the two arms. A simple baseline questionnaire will be administered to all Grade 5 students to collect information on gender-specific risk factors and to evaluate the level of knowledge regarding STH infections. Each child will be weighed and measured (for anthropometric measures), and a stool specimen will be collected and examined for STH eggs (to estimate STH infection rates and intensity). The health hygiene education intervention will begin immediately after the routine deworming program in schools in the intervention arm. Schools in the control arm will follow their standard curriculum. (These schools will be offered the health hygiene education intervention after the 4-month follow-up period.)

Two weeks after the deworming, an efficacy assessment will be undertaken in a sub-sample of 300 previously-infected children (ie. Stool specimens will be collected to estimate cure rates for each STH. This is needed to provide a correction factor in the data analyses.). At the end of the 4-month follow-up study period, the baseline questionnaire will be re-administered to all children in both arms, the children will be weighed and measured, and re-infection rates will be assessed by collecting a stool specimen from each child. Absenteeism rates, based on routine school records, will be assessed from baseline to the end of the follow-up period. An intention-to-treat analysis will be used to compare outcomes in the two arms.

**Study population**

Multidisciplinary and participatory workshops conducted to ascertain local health priorities in the urban slum of Belén demonstrated that intestinal parasites were among the top-five health priorities and that targeted health hygiene education interventions were required [1]. In fact, in a recent survey we conducted in Belén, we found 86% of Grade 5 school children infected by at least one STH species (*Ascaris*=60.4%, *Trichuris*=77.9%, and hookworm=21.2%) [2].

We have obtained current information on the number of children enrolled in Grade 5 in schools of Belén, Peru. For efficiency purposes, only schools with a minimum enrolment of 10 boys and 10 girls in Grade 5 will be considered eligible. Eighteen schools, totalling more than 1438 students, are therefore considered eligible to participate in this trial.

*Inclusion criteria*

- Girls and boys enrolled in Grade 5 in one of the 18 eligible schools in Belén.
- Informed written consent obtained from the parents or legal guardian of the child.
- Verbal assent from child.

*Exclusion criteria*

- Parents (or guardians) refusing participation of their child.
- Child who refuses to participate.


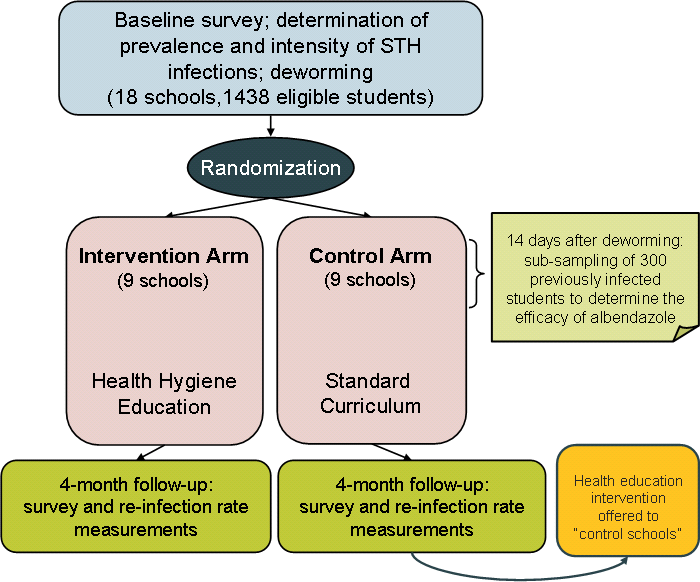


Figure 1: Flowchart of the cluster-randomized controlled trial

**Health hygiene education intervention**

Lack of knowledge on the transmission and prevention methods of STH infections is common factor in affected population groups of Latin America [27]. Therefore, the specific objectives of the health hygiene education intervention are:

i) To implement a gender-specific health hygiene education strategy which will result in a sustainable intervention that encourages the pro-active role of both teachers and students.

ii) To encourage changes in attitudes and practices with the aim of keeping the level of parasite infection low, through increased knowledge.

iii) To identify modifiable gender-specific behavioural and social determinants which favour the acquisition and transmission of STH infections to inform prevention activities.

Workshops will be organized for and by Grade 5 teachers to train them on the health hygiene education strategy. The education material will be provided by the Pan American Health Organization (in Spanish) and will be adapted to the local culture. The health hygiene education intervention will take into account the gender-specific perceptions, values, social constructs, and beliefs of students and teachers relevant to STH infections in order to maximize behavioural changes. After the workshop, Grade 5 teachers will be able to implement the health hygiene education intervention in their classroom and engage their students. Bi-monthly visits with the teacher by our research team and once monthly with the class will be performed to monitor the application of the education program during the 4-month follow-up period. Specifically, the intention of the health hygiene education program is to modify key gender-specific modifiable behavioural determinants that have been shown to be associated with STH infections from our review of the literature:

i) Not using soap for hand washing [28-30]

ii) Not hand washing (no reference to the use of soap) [31-33]

iii) Eating unpeeled or unwashed fruits [31, 34]

iv) Walking barefoot [30, 33]

v) Open air defecation [33-35]

vi) General unhygienic behaviours [36]

**Measurements**

Weight of the child will be measured using a portable flat scale (seca 869, seca corp., Baltimore, MD) and a mobile stadiometer (seca 217, seca corp., Baltimore, MD) will be used to measure their height. Individual risk factors, potential confounding variables, level of knowledge on STH transmission, and behavioural determinants will be measured using an interviewer-administered questionnaire at baseline. After the 4-month follow-up period, the same questionnaire will be re-administered.

Stool specimens will be obtained in small plastic containers at baseline and at the end of the 4-month follow-up period by trained research personnel. Trained technologists will use the Kato-Katz method to examine the specimens for the presence, species and intensity of STH eggs [37]. This technique is the preferred method for assessment of the prevalence and intensity of intestinal parasitic infections in the field [4, 38]. The technologists will be blinded to the group assignment.

Data collection activities during fieldwork will be regularly supervised. Measurement bias will be kept to a minimum by ensuring calibration of study instruments (balance and scale), quality control assessments, and daily review of completed questionnaires. At each assessment, the coordinator will check all completed forms immediately after data collection at the end of each day. Entries on the questionnaires will be checked and if discrepancies or unclear answers appear, a discussion and explanation of the question and eligible answers will take place to avoid making errors the next day. Corrections will be made, if warranted. These types of errors should be kept to a minimum, as there will be a pre-testing phase with all interviewers to ensure correct data collection procedures and records. The consistency of the microscopic readings will be verified both during the examination and afterward as follows. The consistency of egg counting will be evaluated among the laboratory technologists using standard quality control methods [38]. The laboratory supervisor will read 10% of the slides of each microscopist without prior knowledge of the results. In the case of a discrepancy larger than 10%, the two readers will discuss the slides and further slides will be examined to avoid repeated errors. It should be noted that trained microscopists from our previous study will perform the examinations. They have an excellent performance record.

**Sample size justification**

Based on our survey of schools in Belén, 18 schools are eligible and have already demonstrated an interest in participating in the study. They have a total of 1438 students enrolled in Grade 5 (Table 2). Power calculations are based on the formula for logistic regression with a single binary covariate (i.e. the education intervention) and the Wald test was used as the basis for computations [39]. Based on our past parasitological Belén school surveys a response rate of 80.6% is expected [2]. A 5% loss to follow-up is also assumed during the 4-month period. Thus, we estimate a sample size of 1101 participating students, distributed in the 18 schools.

In addition, the power calculations must take into account within-school clustering. The intraclass correlation coefficient (ICC) represents the proportion of the total variance that is accounted for by between-cluster rather than within-cluster variation and can be used to calculate the Effective Sample Size (ESS). Because we could not find any reported ICC of within-school clustering of STH re-infection in the literature, we re-analysed the original database of Casapía et al. [2], which contained the parasitological results of 1074 Belén students, and calculated the within-school ICC of STH prevalence (as an estimation of ICC of re-infection rates). The ICC was calculated using STH prevalence as the outcome and the school as the grouping factor, while adjusting for age, sex, and the school ecological zone (floodplain or upland). The obtained ICC had a value of 0.028 which resulted in an ESS of 412 and an associated design effect of 2.7. Power was estimated assuming that 50% of children will be exposed to the intervention and that the re-infection rate in the control group will be 49.5%. The latter is based on the reported 3-month re-infection rate of Malaysian school-children treated with albendazole [40]. Thus, the proposed study has good power (80.3%) to detect a moderate effect (i.e., OR=1.75) and has an excellent power (93.4%) to detect a strong effect, corresponding to an OR of 2.00.

The efficacy of the deworming program will be determined using a sub-sample of 300 previously infected children. Assuming the most conservative value of a 50% cure rate, this sample size of 300 will result in precise species-specific cure rates estimated with a maximum 95% confidence interval of ± 5.7%. No within-school clustering is assumed for cure rates because variables affecting cure rates are intrinsic to the parasites and the hosts themselves, not the schools.

Table 2: Number of Grade 5 students enrolled in 2007 and 2008 in the 18 eligible schools located in the Belén district of Iquitos, Peruvian Amazon

| **School name** | **Nb. grade 5 students 2007** | **Nb. grade 5 students 2008** | **Nb. classrooms** |
| --- | --- | --- | --- |
| Bagazan | 42 | 49 | 2 |
| Santo Cristo Bagazan | 134 | 125 | 4 |
| Diego Natal Juan | 109 | 87 | 3 |
| Ruy Guzmán Hidalgo | 181 | 160 | 5 |
| Participación | 147 | 116 | 3 |
| Enry Herve Linares Soto | 26 | 24 | 1 |
| Ramón Castilla | 266 | 203 | 6 |
| Sara Alicia Saberbein Pinedo | 175 | 186 | 5 |
| Sachachorro | 65 | 56 | 2 |
| San Lucas | 104 | 113 | 3 |
| Violeta Correa | 47 | 53 | 2 |
| Sagrada Familia | 53 | 79 | 2 |
| Isla Iquitos | 34 | 32 | 1 |
| San José | 43 | 25 | 1 |
| San Francisco | 43 | 42 | 2 |
| Nuevo Liberal | 63 | 37 | 1 |
| La Victoria de Jesús | 33 | 31 | 2 |
| Nuevo Campeón | 25 | 20 | 1 |
| **Total** | **1590** | **1438** |  |

**Planned statistical analyses**

All children in both arms will be included in the analysis (i.e., an intent-to-treat analysis). Preliminary analyses will include descriptive statistics (means and standard deviations for continuous variables; frequencies for categorical variables) for all measurements in each intervention group separately. Gender-disaggregated data will be presented in tables, whenever pertinent.

Multivariate analyses will be used to compare the post-deworming (at 4-months) STH re-infection rates between the two groups. To account for the within-school clustering, hierarchical (random effects) logistic regression models will be used [41]. The model will include important confounding variables such as gender, sex, age, school ecological zone, as well as any baseline characteristics that may not be balanced between the two groups. Subgroup analysis will be performed for boys and girls separately as well as for each of the three helminth species studied (*Ascaris*, *Trichuris*, and hookworm).

All statistical tests will be two-sided and a 0.05 level of significance will be used throughout for determination of statistical significance. Point estimates of the intervention effect with 95% confidence intervals will be presented to demonstrate the precision of the study results.

**Safety reporting**

As this study is testing the impact of an educational intervention on selected non-invasive outcome measures, no adverse events or complications associated with this intervention are expected. We consider that the administration of the questionnaire, the health hygiene education intervention, and the collection of the stool specimen will have no safety effect.

With respect to the routine deworming program, it may be that the deworming pill has a minor transitory side effect. The proposed anthelmintic, albendazole, has an excellent safety record involving over billions of doses [7, 42]. This drug is considered very safe and its administration is not a concern. Mass deworming of school children with albendazole is recommended by WHO, UNICEF, and the World Bank, among other organizations. Because of its overwhelmingly benefits, in 2001, The World Health Assembly unanimously ratified the resolution that deworming should reach at least 75% of all school children in endemic areas by the year 2010. Nevertheless, we will ensure that the local health authorities are informed about our deworming activities. We have collaborated with them in the past and will continue to do so throughout this research.

**Anticipated benefits**

It is anticipated that all schools and students participating in the research will benefit from this study. Schools will benefit directly because the capacity-building workshops and the education intervention program will provide them with new knowledge and tools to address STH infections, and indeed, other health hygiene concerns. Students participating in the study will also benefit directly from the study because they will be given the opportunity of increased knowledge. School absenteeism is expected to be reduced, benefiting students, schools, families and their community. As our previous study demonstrated, 86% of grade 5 students in Belén are infected by at least one parasites species [2]. Participation in this study will therefore contribute to improved health status.

**Potential risks**

As the planned intervention will consist of a health hygiene education program, there are no associated risks. There are also no associated risks with the data collection procedures (questionnaires and provision of stool specimens).

**Privacy and confidentiality**

Participants and schools will be assigned an identification number for the duration of the study to ensure the confidentiality of any results. All original documents, including questionnaires and informed consent forms, will be kept (in Peru) in a locked cabinet and room. All electronic information, such as databases, will be stored on a password-protected computer in a locked room. Access to original documents and electronic information will initially be restricted to the Project Director, Principal Investigator and study coordinators only.

**Details of trial team**

The trial management on site in Iquitos (Peru) will be under the local and daily supervision of Dr. Martín Casapía (Project Director), together with the study coordinator, Salomé Chapiama. Ms. Chapiama is trained as a nurse and has collaborated on more than seven research projects with us. She is an excellent coordinator and has the confidence of the investigator team. The Canadian members of the research teams include Dr. Theresa Gyorkos (principal investigator) and her research coordinator, Mathieu Maheu-Giroux. M. Maheu-Giroux will also be present in Iquitos during the implementation phase and randomization phase of the trial. He holds two Master’s degrees from McGill University (Ecology and Epidemiology) and has already conducted research in Iquitos. Dr. Theresa Gyorkos is responsible for the overall study design including all methodological issues. Although involved at a distance, all study decisions will be taken in consultation with the entire team. Dr. Gyorkos has been working on a variety of research projects in this region of Peru over the last 8 years and has strong ties with local collaborators and health officials. Furthermore, she has already conducted a large RCT in this region of Peru. This is an international collaboration of Canadian and Peruvian researchers who have had extensive experience in conducting epidemiological research together.

The two major collaborating centres are the Research Institute of the McGill University Health Centre in Montreal (Canada) and the Asociación Civil Selva Amazónica in Iquitos (Peru).

**Ethical oversight**

The principal investigator, Project Director, study coordinators and all other research personnel will conduct the study in an ethical manner which is consistent with the international principles of good practice and will function according to institutional policies of the McGill University Health Centre and the Asociación Civil Selva Amazónica, governing human subjects’ research, applicable research guidelines and in compliance with the law. The research investigators understand that the study is subject to review and approval from both the Research Ethics Board in Canada and the Dirección de Salud de Loreto in Peru.

The trial will be registered according to international regulations.

**Compensation**

No financial or other incentives or compensation will be given to any study participant. At the end of the trial, participating schools will keep all educational materials used. The principal investigator and project director will also facilitate access to additional educational materials, as requested by the schools.

**Results dissemination**

The results of this trial will be published and presented in national and international fora, including scientific conferences and peer-reviewed journals. Dissemination in local, regional and national media in Peru will also be explored. The results will be of interest to all 129 other STH-endemic countries who are currently, or who are planning to, implement a school-based deworming program. Therefore, our results will be sent to newsletters such as Action Against Worms, the Deworm the World initiative, the Partners for Parasite Control, etc.

**Funding**

This study is supported by an Interdisciplinary Capacity Enhancement grant from the Canadian Institutes of Health Research (Dr. Theresa W. Gyorkos). Funding continues to 2011.

**Chronogram of research activities**

The following table provides the detailed schedule of all research activities, with approximate start and end dates.

|  | **2009** | | **2010** | | | | | | | | | | | |
| --- | --- | --- | --- | --- | --- | --- | --- | --- | --- | --- | --- | --- | --- | --- |
| **Activities** | November | December | January | February | March | April | May | June | July | August | September | October | November | December |
| Preliminary school visits | ✓ | ✓ |  |  |  |  |  |  |  |  |  |  |  |  |
| Ethics approval (Canada and Peru) |  | ✓ | ✓ | ✓ |  |  |  |  |  |  |  |  |  |  |
| Trial registration |  |  |  | ✓ |  |  |  |  |  |  |  |  |  |  |
| Informing health authorities; meeting all school principals |  |  |  | ✓ |  |  |  |  |  |  |  |  |  |  |
| Randomization |  |  |  | ✓ |  |  |  |  |  |  |  |  |  |  |
| Training workshops for teachers in the intervention schools |  |  |  | ✓ | ✓ |  |  |  |  |  |  |  |  |  |
| Organization of meetings with parents (informed consent) |  |  |  |  | ✓ |  |  |  |  |  |  |  |  |  |
| Baseline survey (questionnaire, weight and height, stool specimen) – before deworming |  |  |  |  |  | ✓ |  |  |  |  |  |  |  |  |
| Deworming |  |  |  |  |  | ✓ |  |  |  |  |  |  |  |  |
| Start of the education program in the intervention schools |  |  |  |  |  | ✓ | ✓ | ✓ | ✓ | ✓ |  |  |  |  |
| Cure rate estimation (2 weeks post-deworming) |  |  |  |  |  |  | ✓ |  |  |  |  |  |  |  |
| Second survey (questionnaire, weight and height, stool specimen) |  |  |  |  |  |  |  |  |  | ✓ |  |  |  |  |
| Training workshop of the education program offered to control schools |  |  |  |  |  |  |  |  |  | ✓ |  |  |  |  |
| Health education program offered to control schools |  |  |  |  |  |  |  |  |  | ✓ | ✓ | ✓ | ✓ |  |
| Data analysis |  |  |  |  |  |  |  |  |  |  | ✓ | ✓ | ✓ |  |
| Begin dissemination of results |  |  |  |  |  |  |  |  |  |  |  |  |  | ✓ |

**References**

1 Casapía M, Joseph SA, Gyorkos TW. Multidisciplinary and participatory workshops with stakeholders in a community of extreme poverty in the Peruvian Amazon: Development of priority concerns and potential health, nutrition and education interventions. *International Journal for Equity in Health* 2007;**6**:6-14.

2 Casapía M, Joseph SA, Núñez C*, et al.* Parasite risk factors for stunting in grade 5 students in a community of extreme poverty in Peru. *International Journal for Parasitology* 2006;**36**:741-7.

3 Hotez PJ, Bundy DAP, Beegle K*, et al.* Helminth Infections: Soil-Transmitted Helminth Infections and Schistosomiasis. In: Jamison DT, Breman JG, Measham AR*, et al.*, eds. *Disease Control Priorities in Developin Countries*. Washington DC & New York, NY: Oxford University Press and the World Bank 2006:1352.

4 WHO. Prevention and Control of Schistosomiasis and Soil-Transmitted Helminthiasis. Geneva, Switzerland: Report of a WHO Expert Committee, Technical Report Series, No 912 - World Health Organization 2002:63.

5 PAHO. *Marco de referencia de un programa regional para el control de las geohelmintosis y esqustosomosis en América*. Santo Domingo, República Dominicana: Organización Panamericana de la Salud 2003.

6 Albonico M, Montresor A, Crompton DW*, et al.* Intervention for the control of soil-transmitted helminthiasis in the community. *Advances in Parasitology* 2006;**61**:311-48.

7 WHO. *Preventive chemotherapy in human helminthiasis - Coordinated use of anthelminthic drugs in control interventions: a manual for health professionals and programme managers*. Geneva, Switzerland: World Health Organization 2006.

8 Crompton DW. The public health importance of hookworm diseases. *Parasitology* 2000;**121**:39-50.

9 O'Lorcain P, Holland CV. The public health importance of *Ascaris lumbricoides*. *Parasitology* 2000;**121**:51-71.

10 Bethony J, Brooker S, Albonico M*, et al.* Soil-transmitted helminth infections: ascaris, trichuriasis, and hookworm. *The Lancet* 2006;**367**:1521-32.

11 Montresor A, Crompton DWT, Gyorkos TW*, et al.* Helminth Control in School-Age Children. Geneva, Switzerland: World Health Organization 2002:64.

12 Bundy DAP, Wong MS, Lewis LL*, et al.* Control of geohelminths by delivery of targeted chemotherapy through schools. *Transactions of the Royal Society of Tropical Medicine and Hygiene* 1990;**84**:115-20.

13 The Lancet. Thinking beyond deworming. *The Lancet* 2004;**364**:1993-4.

14 WHO. Strategy Development and Monitoring for Parasitic Diseases and Vector Control Team. Deworming: the Millennium Development Goals. The evidence is in: deworming helps meet the Millennium Development Goals. Geneva, Switzerland: WHO/CDS/CPE/PVC/ 2005:12.

15 Annual report of the council of economic advisers. Economic report of the President - Chapter 6. Washington, DC: Transmitted to the Congress 2003:241.

16 Awasthi S, Pande VK, Fletcher RH. Effectiveness and cost-effectiveness of albendazole in improving nutritional status of pre-school children in urban slums. *Indian Pediatrics* 2000;**37**:19-29.

17 Albonico M, Smith PG, Ercole E*, et al.* Rate of reinfection with intestinal nematodes after treatment of children with mebendazole or albendazole in a highly endemic area. *Transactions of the Royal Society of Tropical Medicine and Hygiene* 1995;**89**:538-41.

18 The Partnership for Child Development. The cost of large-scale school health programmes which deliver anthelmintics to children in Ghana and Tanzania. *Acta Tropica* 1999;**73**:183-204.

19 Asaolu SO, Ofoezie IE. The role of health education and sanitation in the control of helminth infections. *Acta Tropica* 2003;**86**:283-94.

20 Luong TV. De-worming school children and hygiene intervention. *International Journal of Environmental Health Research* 2003;**13**:S153-S9.

21 Lansdow R, Ledward A, Hall A*, et al.* Schistosomiasis, helminth infection and health education in Tanzania: achieving behaviour change in primary schools. *Health Education Research* 2002;**17**:425-33.

22 Long-Shan X, Bao-Jun P, Jin-Xiang L*, et al.* Creating health-promoting schools in rural China: a project started from deworming. *Health Promotion International* 2000;**15**:197-206.

23 Albright JW, Basaric-Keys J. Instruction in behavior modification can significantly alter soil-transmitted helminth (STH) re-infection following therapeutic de-worming. *Southeast Asian Journal of Tropical Medicine and Public Health* 2006;**37**:48-57.

24 Hadidjaja P, Bonang E, Suyardi MA*, et al.* The effect of intervention methods on nutritional status and cognitive function of primary school children infected with *Ascaris lumbricoides*. *American Journal of Tropical Medicine and Hygiene* 1998;**59**:791-5.

25 Albonico M, Shamlaye N, Shamlaye C*, et al.* Control of intestinal parasitic infections in Seychelles: a comprehensive and sustainable approach. *Bulletin of the World Health Organization* 1996;**74**:577-86.

26 Anantaphruti MT, Waikagul J, Maipanich W*, et al.* School-based health education for the control of soil-transmitted helminthiases in Kanchanaburi province, Thailand. *Annals of Tropical Medicine and Parasitology* 2008;**102**:521-8.

27 Botero D. Persistencia de parasitosis intestinales endémicas en América Latina. *Boletín de la Oficina Sanitaria Panamericana* 1981;**90**:39-47.

28 Olsen A, Samuelsen H, Onyango-Ouma W. A study of risk factors for intestinal helminth infections using epidemiological and anthropological approaches. *Journal of Biosocial Sciences* 2001;**33**:569-84.

29 Fung IC, Cairncross S. Ascariasis and handwashing. *Transactions of the Royal Society of Tropical Medicine and Hygiene* 2009;**103**:215-22.

30 Parajuli RP, Umezaki M, Watanabe C. Behavioral and nutritional factors and geohelminth infection among two ethnic groups in the Terai region, Nepal. *American Journal of Human Biology* 2009;**21**:98-104.

31 Hohmann H, Panzer S, Phimpachan C*, et al.* Relationship of intestinal parasites to the environment and to behavioral factors in children in the Bolikhamxay province of Lao PDR. *Southeast Asian Journal of Tropical Medicine and Public Health* 2001;**32**:4-13.

32 Corrales LF, Izurieta R, Moe CL. Association between intestinal parasitic infectionas and type of sanitation system in rural El Salvador. *Tropical Medicine & International Health* 2006;**2**:1821-31.

33 Gamboa MI, Kozubsky LE, Costas ME*, et al.* Associations between geohelminths and socioenvironmental conditions among different human populations in Argentina [in Spanish]. *Revista Panamericana de Salud Pública / Pan American Journal of Public Health* 2009;**26**:1-8.

34 Wördemann M, Polman K, Menocal Heredia LT*, et al.* Prevalence and risk factors of intestinal parasites in Cuban children. *Tropical Medicine & International Health* 2006;**11**:1813-20.

35 Bóia MN, Carvalho-Costa FA, Sodré FC*, et al.* Mass treatment for intestinal helminthiasis control in an Amazonian endemic area in Brazil. *Revista do Instituto de Medicina Tropical de São Paulo* 2006;**48**:189-95.

36 Albright JW, Hidayati NR, Basaric-Keys J. Behavioral and hygienic characteristics of primary schoolchildren which can be modified to reduce the prevalence of geohelminth infections: a study in central Java, Indonesia. *Southeast Asian Journal of Tropical Medicine and Public Health* 2005;**36**:629-40.

37 Katz N, Chavez A, Pellegrino J. A simple device for quantitative stool thick-smear technique in *Schistosomiasis mansoni*. *Revista do Instituto de Medicina Tropical de São Paulo* 1972;**14**:397-400.

38 Montresor A, Crompton DW, Bundy DAP*, et al.* Guidelines for the Evaluation of Soil-Transmitted Helminthiasis and Schistosomiasis at Community Level. Geneva, Switzerland: World Health Organization 1998:45.

39 Demindenko E. Sample size determination for logistic regression revisited. *Statistics in Medicine* 2007;**26**:3385-97.

40 Al-Mekhlafi MH, Surin J, Atiya AS*, et al.* Pattern and predictors of soil-transmitted helminth reinfection among aboriginal schoolchildren in rural Peninsular Malaysia. *Acta Tropica* 2008;**107**:200-4.

41 Gelman A, Hill J. *Data Analysis using Regression and Multilevel/Hierarchical Models*. New York, NY: Cambridge University Press 2007.

42 Albonico M, Allen H, Chitsulo L*, et al.* Controlling soil-transmitted helminthiasis in pre-school-age children through preventive chemotherapy. *PLoS Neglected Tropical Diseases* 2008;**2**:e126.

43 Albonico M, Engels D, Savioli L. Monitoring drug efficacy and early detection of drug resistance in human soil-transmitted nematodes: a pressing public health agenda for helminth control. *International Journal of Parasitology* 2004;**34**:1205-10.

**Annexes**

**Annex 1**

Informed Consent Form - Parents (Spanish Version)

**Annex 2**

Informed Consent Form - Parents (English Version)

**Annex 3**

Informed Assent Form – Students (Spanish Version)

**Annex 4**

Informed Assent Form – Students (English Version)

**Annex 5**

Questionnaire (Spanish Version)

**Annex 6**

Questionnaire (English Version)

| Consentimiento Informado - Padres |
| --- |

**Annex 1:** Informed Consent Form - Parents (Spanish Version)

**Efectividad de una intervención educativa, post desparasitación, para reducir las lombrices y la inasistencia en los escolares de 5^to^ grado, Belén**


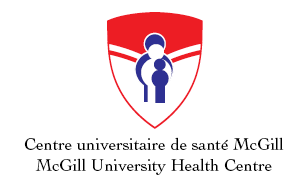

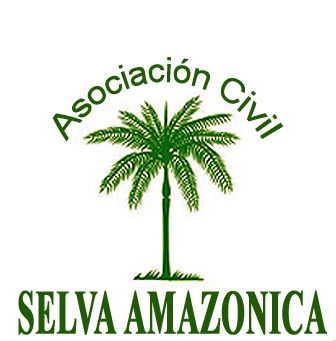

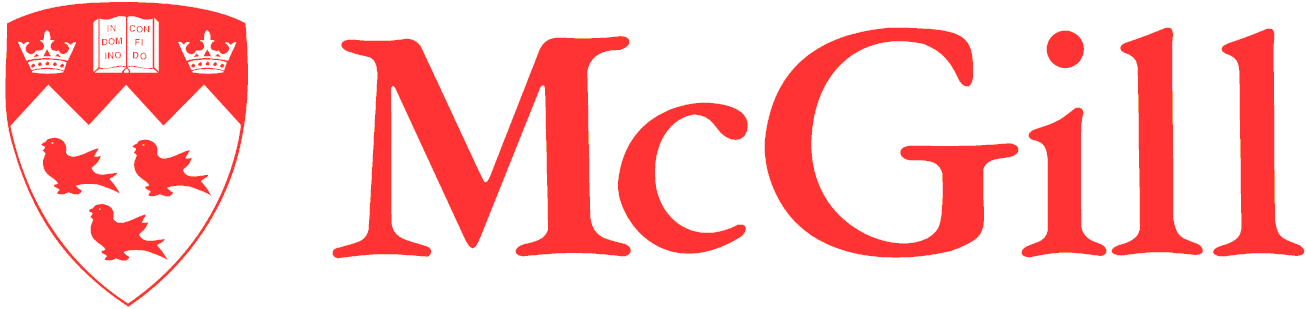


**Investigadores e Instituciones**

***Canadá***: Dra. Theresa W. Gyorkos (Investigadora Principal, Centro de Salud de la Universidad McGill - Instituto de Investigaciones y Universidad McGill); Mathieu Maheu-Giroux (Coordinador, Centro de Salud de la Universidad McGill - Instituto de Investigaciones).

***Perú***: Dr. Martín Casapía (Director del Proyecto, Asociación Civil Selva Amazónica); Lic. Enf. Salomé Chapiama (Coordinadora, Asociación Civil Selva Amazónica).

**Introducción**

Les invitamos a usted y a su hijo a participar en un programa de desparasitación y educación sanitaria del Dr. Martín Casapía de la Asociación Civil Selva Amazónica (ACSA) y de la Dra. Theresa Gyorkos del Centro de Salud de la Universidad McGill (CSUM). Este tipo de programa de desparasitación basado en las escuelas, en los cuales se administra un purgante a los escolares, es recomendado por la Organización Mundial de la Salud en muchos países y, también, tiene el apoyo del Ministerio de Salud del Perú.

Antes de que decida participar, es importante que comprenda los contenidos de este formato de consentimiento, los riesgos y beneficios para tomar una decisión informada, y que haga cualquier pregunta si algo no entiende. Por favor, lea este formato de consentimiento informado y tómese el tiempo para que tome una decisión. Si decide participar en este estudio, le pedimos firme este formato.

**Propósito del estudio**

El propósito de este estudio es de medir el impacto de un programa de desparasitación y educación sanitaria en los escolares de 5^to^ grado de Belén. Hay muchos componentes en un programa de desparasitación y nos gustaría averiguar si la integración de una estrategia de educación sanitaria en dicho programa es un componente importante. En la eventualidad que esta estrategia de educación sea importante, vamos a ayudar a las escuelas de Belén para ofrecerla a sus niños después del estudio.

**Procedimiento del estudio**

Si acepta participar, le pediremos autorización para reunirnos con su hijo(a) (que estudia en 5^to^ grado) en tres ocasiones, durante los próximos 4 meses, y cada reunión tendrá una duración de aproximadamente 15 minutos.

1) Primero, tomaremos medidas de peso y altura. Haremos algunas preguntas a su hijo(a) acerca de su salud y hábitos. Pediremos también que su hijo(a) nos proporcione una muestras de heces para buscar lombrices (parásitos intestinales).

2) Segundo, administraremos una pastilla antiparasitaria (400 mg albendazol) con papaya y un jugo de fruta. En el caso que su hijo(a) esté infectado por lombrices, regresaremos después de dos semanas para averiguar si la pastilla funciono.

3) Finalmente, a los 4 meses de la primera visita, volveremos a la escuela y haremos otras preguntas a su hijo(a) acerca de su salud, lo vamos a pesar y medir, y pedirle otra muestra de heces.

No podemos empezar con la estrategia de educación sanitaria en todas las escuelas al mismo tiempo. Entonces, empezaremos con la intervención de educación en la mitad de las escuelas al mismo tiempo que la desparasitación. En la otra mitad de las escuelas se ejecutara la estrategia de educación después de los 4 meses de seguimiento.

**Asuntos de Ética**

Le informamos que este estudio fue revisado y aprobado por la Oficina de Ética del Centro de Salud de la Universidad McGill en Montreal, Canadá. También, fue aprobado por el Comité Institucional de Bioética (Impacta, Perú) y el Director de la Institución Educativa de su hijo(a).

**Riesgos**

No hay ninguno riesgo previsible asociado al programa de educación sanitaria. El suministro de una dosis de la pastilla antiparasitaria, albendazol, es muy segura y efectiva y se da a los escolares de mucho países. La mayoría de esos escolares no tiene ninguna reacción colateral, pero se nota síntomas menores en una minoría de ellos. Además, esos efectos secundarios (dolor abdominal, diarrea, mareos, etc.) son transitorios y desaparecen en las 48 horas siguientes al suministró del antiparasitario [43]. Por eso, la Organización Mundial de la Salud y la Organización panamericana de salud opinan que el albendazol es muy seguro y recomiendan el suministro de esta pastilla a las poblaciones que viven en las zonas endémicas.

**Beneficios Potenciales**

Estudios previos en la zona demostraron que la mayoría (86%) de los niños de 5^to^ grado matriculados en las escuelas de Belén se infecta por lo menos con una de las tres especies de lombrices (parásitos intestinales). Esos parásitos causan retrasos e impedimentos en el crecimiento y desarrollo físico e intelectual de los niños afectados. El beneficio directo que tendrá su hijo de participar en el estudio es la posibilidad de curarse de esas lombrices y mejorar su salud. Además, usaremos la información del estudio para respaldar la desparasitación continuada en las escuelas de Belén. Eso quiere decir que hablaremos con las autoridades de salud y educación para que incluyan un programa de desparasitación cada año.

**Confidencialidad**

Toda la información obtenida durante este estudio será mantenida en estricta confidencialidad. Su nombre y el nombre de su hijo quedarán anónimos para todas las personas que no son integrantes del equipo de investigación y se pondrá toda la información en un fichero cerrado en la oficina del investigador con acceso limitado. Solo el equipo de investigación podrá tener acceso a la información, después de haber obtenido el permiso escrito de los investigadores principales. Los resultados de este estudio pueden ser publicados pero los nombres de los participantes no serán publicados. Su identidad no será revelada en los resultados combinados. Para verificar los datos de la investigación, el Oficial de Garantía de Calidad de la Junta de Ética de Investigaciones del CSUM puede examinar estos archivos. Firmando este consentimiento informado, usted nos da la autorización de proporcionar la información relacionada a su participación al Oficial de Garantía de Calidad de la Junta de Ética de Investigaciones del CSUM, el Comité institucional de Bioética de la Asociación Civil Impacta Salud y Educación, y el juez.

**Participación voluntaria y retirada de este estudio**

La participación de su hijo en este estudio es estrictamente voluntaria. Usted puede negar la participación de su hijo(a) en el estudio y puede suspender su participación en cualquier momento sin explicación, y sin pena o pérdida de beneficios a los que tiene derecho. En el caso que usted suspenda su participación, su hijo(a) no será afectado en cuanto a su atención medica o su participación en estudios de investigación futuros. Usted recibirá toda la información que pueda comprometer la voluntad suya o de su hijo(a) a seguir participando en el presente estudio.

**Costos y compensación**

No hay ningún costo asociado a su participación en el estudio. Su hijo(a) recibirá una dosis de albendazol en la escuela. La pastilla es gratuita. No le ofreceremos compensación a su hijo(a) o a usted por participar en este estudio. La escuela de su hijo(a) recibirá material educativo en relación a las lombrices (parásitos intestinales).

**Persona de contacto**

Si tiene alguna pregunta acerca del estudio, puede contactar con la Coordinadora del Proyecto, Lic. Enf. Salomé Chapiama a 965-939153, línea gratuita 0800-1321, y gustosamente se le responderá.

Si su hijo(a) sufre algún síntoma que pueda guardar relación con este estudio, comuníquese con la Dra. Lilian Saavedra al 065-236277 o al 065-965 621863. También puede presentarse a la oficina de la Asociación Civil Selva Amazónica, cual dirección es Urb. Jardín N 27, Fanning 4^ta^ Cuadra, Iquitos.

Si tiene alguna pregunta sobre los derechos de su hijo(a) como participante de una investigación, o alguna duda o inquietud sobre el tratamiento que recibirá en este estudio, comuníquese con el Dr. Andrés Paredes. Presidente Interino del Comité de Bioética de la Asociación Civil Impacta Salud y Educación al teléfono 01242-3072 anexo 130.

Declaración de consentimiento

He comprendido los contenidos de este formato de consentimiento Informado, y estoy de acuerdo en participar en este estudio de investigación. He tenido la oportunidad de hacer preguntas durante una sesión de información y todas mis preguntas han sido resueltas a mi satisfacción. He tenido tiempo suficiente para considerar la información antes mencionada y pedir consejos si elijo hacerlo. Firmando este formulario de consentimiento, no estoy renunciando a mis derechos legales.

__________________________________

Nombre de niño/a

____________________ _________________________ ___________________

Nombre de padre/tutor Firma de padre/tutor Fecha (dd/mm/aaaa)

____________________ _________________________ ___________________

Nombre de testigo Firma de testigo Fecha (dd/mm/aaaa)

| Informed Consent –  Parents |
| --- |

**Annex 2:** Informed Consent Form - Parents (English Version)

Effectiveness of a post-deworming education intervention to reduce infection and absenteeism in grade 5 school children


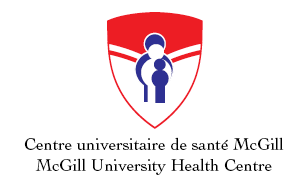

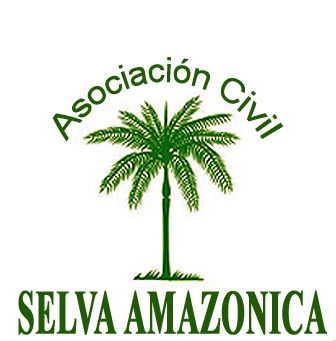

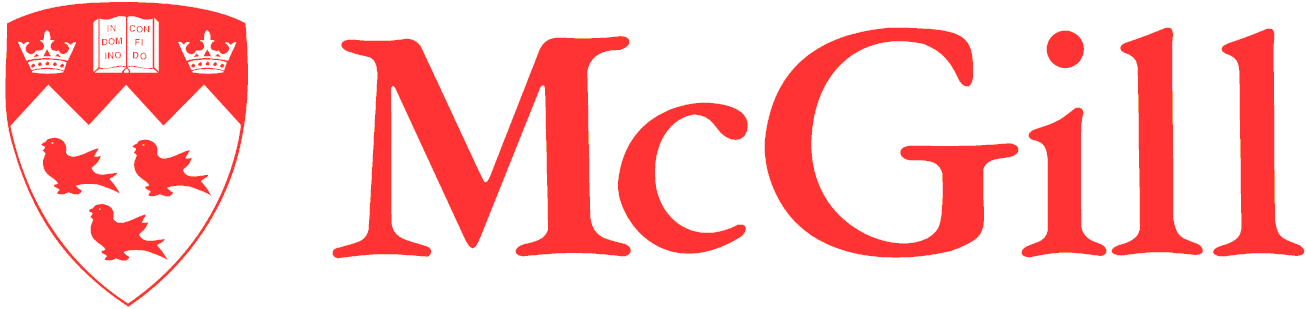


**Investigators and Institutions**

***Canada***: Dr. Theresa W. Gyorkos (Principal Investigator, Research Institute of the McGill University Health Centre - McGill University); Mathieu Maheu-Giroux (Coordinator, Research Institute of the McGill University Health Centre).

***Perú***: Dr. Martín Casapía (Project Director, Asociación Civil Selva Amazónica); Salomé Chapiama (Coordinator, Asociación Civil Selva Amazónica).

**Introduction**

We are inviting you and your child to participate in an educational deworming program being conducted by Dr. Casapía from the Asociación Civil Selva Amazónica (ACSA) and Dr. Theresa Gyorkos from the McGill University Health Centre (MUHC) in Canada. This type of deworming program in schools, where a deworming drug is given to schoolchildren, is recommended by the World Health Organization in many countries and is also supported by the Ministry of Health of Peru.

Before you decide to participate, it is important that you understand the contents of this consent form, the risks and benefits to make an informed decision, and ask any questions if there is anything that you do not understand. Please read this entire consent form and take your time to make a decision. If you decide to participate in this study, you will be asked to sign this informed consent form.

**Purpose of the study**

The purpose of this study is to assess the impact of a deworming educational program in Grade 5 children attending primary schools in Belén. There can be many components to a deworming program and we would like to study whether specific health education is an important component. If it is, then we will help Belen schools to offer this health education to your children after our study.

**Study procedures**

If you agree to participate, you will be asked to grant us permission to meet your child (who is in Grade 5) on three occasions, within the next 4 months, and each time we will take about 15 minutes of his/her time.

1) We will first take your child’s weight and height measurements. We will ask your child some questions about his/her health and health habits. We will also ask your child to provide us with a stool specimen at school to check for intestinal parasites.

2) Second, we will give your child a deworming pill (400 mg. albendazole) together with some papaya and fruit juice. If your child is infected with intestinal parasites, he or she may be asked to give us another stool specimen after two weeks, to check if the pill worked.

3) Finally, 4 months after the first visit, we will return to your child’s school and ask him/her to be measured again for weight and height, to give us another stool specimen and to answer some questions about his/her health and health habits.

We cannot start everything in all 18 schools in Belen at the same time. Therefore, in half of the schools, we will begin the health education program at the same time as the deworming, and in the other half of the schools, we will begin the health education program approximately 4 months after the deworming.

**Ethical issues**

We would like to assure you that this study has been reviewed and has received ethics approval from the Ethics Review Board at the McGill University Health Centre in Montreal, Canada. In addition, it has been approved by the Ministry of Health in Loreto and the principal at your child’s school.

**Risks and discomforts**

There are no foreseeable risks associated with this educational program. The deworming pill, albendazole, is very safe and effective and is being given to school children in many parts of the world. Most children do not have any side effects at all after deworming, but some children complain of minor symptoms. If present at all (eg. abdominal pain, diarrhea, nausea, etc.), they are temporary and usually disappear within 48 hours. PAHO and the World Health Organization consider albendazole very safe and in fact recommend that it be given to school children where parasites are common.

**Potential benefits**

A previous study conducted in schools of Belén showed that the majority (86%) of Grade 5 students are infected with at least one species of intestinal parasites. These parasites can cause delays and impairment in the physical and intellectual growth and development of your child. The direct benefit that will be gained by your child by participating in this study is that he/she will be treated for these intestinal parasites. This will improve his/her health.

Furthermore, we will use the information from this study to support continued deworming in Belen schools. This means that we will speak with health and education authorities about including deworming every year.

**Confidentiality**

All information obtained during this study will be kept strictly confidential. Your name and the name of your child will not be given to anyone outside of the research team and the information will be locked in a filing cabinet in the investigator’s office. Only the research team will have access to the information and only after first receiving the approval of the principal investigator. The results of this study will be published but no names will be used at any time. Your identity will not be revealed in the combined results. In order to verify the research data, the Quality Assurance Officer from the MUHC Research Ethics Boards may review these records. By signing this consent form, you give us permission to release information regarding your participation in this study to these individuals.

**Voluntary participation and withdrawal from this study**

The participation of your child in this study is strictly voluntary. You may refuse to have your child participate or you may discontinue his/her participation at any time without explanation, and without penalty or loss of benefits to which you are otherwise entitled. If you discontinue your child's participation, your child will suffer no prejudice regarding the medical care or participation in any other research study. You will be informed of any new findings that may affect your child's willingness to continue your participation.

**Costs and compensation**

There are no costs associated with your participation in this study. Your child will receive one dose of albendazole at school. This will be free. Neither you nor your child will be given any money to participate in this study. Your child's school will receive some teaching materials during the study.

**Contact persons**

If you have any questions regarding the study, you can contact the Project Coordinator, Lic. Enf. Salomé Chapiama at 965-939153 (cell), 221341 anexo 151 (landline) or 0800-1321 (toll free).

If your child develops any symptoms related to this study, please communicate with Dr Lilian Saavedra at 065-236277 or 065-965 621863. You can also present yourself to the office of the Asociación Civil Selva Amazónica, which address is Urb. Jardín N 27, Fanning 4^ta^ Cuadra, Iquitos.

If you have any questions regarding the right of your child as a participant en this study, or any other concern regarding the deworming drug, please call Dr. Andrés Paredes, president of the ‘Comité de Bioética de la Asociación Civil Impacta Salud y Educación’ at 01242-3072 extension 130.

**Declaration of consent**

I understand the contents of this consent form, and I agree that my child participates in this research study. I have had the opportunity to ask questions in an information session and all my questions have been answered to my satisfaction. I have been given sufficient time to consider the above information and to seek advice if I choose to do so. By signing this consent form, I am not giving up any of my legal rights.

______________________________________________

Child’s name

______________________ ___________________________ __________________

Parent’s/guardian’s name Parent’s/guardian’s signature Date (dd/mm/yyyy)

______________________ ___________________________ __________________

Witness’ name Witness’ signature Date (dd/mm/yyyy)

**Annex 3:** Informed Assent Form - Students (Spanish Version)

| **Asentimiento Informado - Niños** |
| --- |

**Efectividad de una intervención educativa, post desparasitación, para reducir las lombrices y la inasistencia en los escolares de 5^to^ grado, Belén**


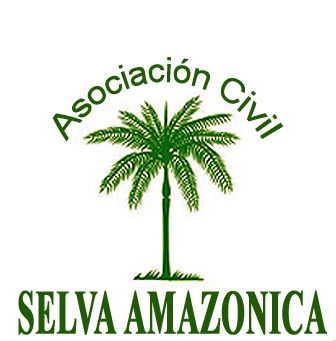

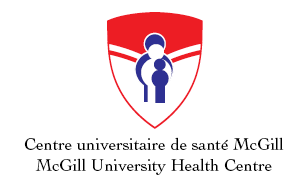

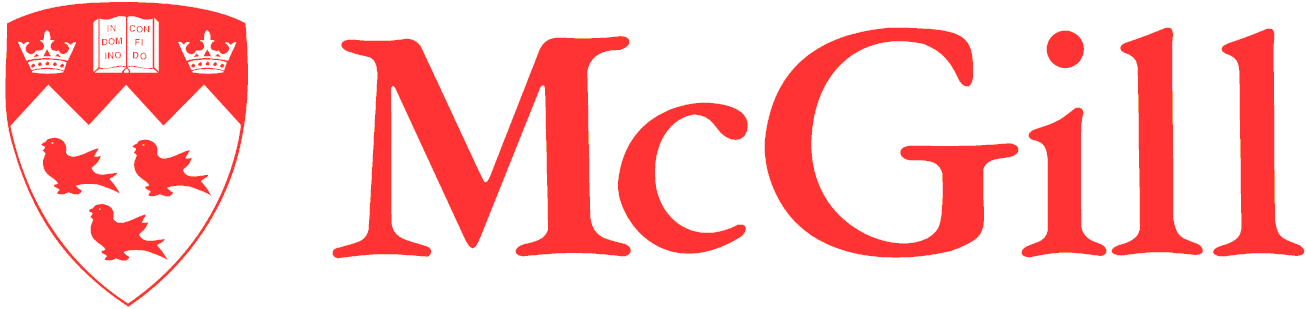


**Investigadores e Instituciones**

***Canadá***: Dra. Theresa W. Gyorkos (Investigadora Principal, Centro de Salud de la Universidad McGill - Instituto de Investigaciones y Universidad McGill); Mathieu Maheu-Giroux (Coordinador, Centro de Salud de la Universidad McGill - Instituto de Investigaciones).

***Perú***: Dr. Martín Casapía (Director del Proyecto, Asociación Civil Selva Amazónica); Lic. Enf. Salomé Chapiama (Coordinadora, Asociación Civil Selva Amazónica).

Introducción

Tus padres me dieron la autorización para hablar contigo sobre un proyecto que trabajo con mis colegas. El proyecto trata de las lombrices (parásitos intestinales) que viven dentro de tu cuerpo y de cómo podemos eliminarlas. Ahora, voy a explicarte con más detalle en qué consiste este proyecto y después te preguntaré si quieres participar en el proyecto.

**¿Porque estamos aquí?**

Queremos hablarte de un estudio que involucra a niños(as) como tú en los centros educativos de Belén. Queremos saber si estas interesado en participar en el estudio. Buscamos medidas para eliminar las lombrices (parásitos intestinales) que dañan tu salud.

**¿Qué ocurrirá si participas en el estudio?**

Si aceptas participar en el estudio, te preguntaremos algunas cosas acerca de tus hábitos y de tu salud. Encontraras a uno de nuestros entrevistadores. Vamos a pesarte y tallarte. También, te pediremos que nos proporciones una pequeña muestra de tus heces para examinar si tienes lombrices. Después, te daremos una pastilla (antiparasitario) para curarte de las lombrices, con una fruta y un jugo. Volveremos entre una y dos veces para tomar otras muestras de heces y averiguar que no estás infectado por las lombrices. Mientras que hagas estas cosas, todo lo que te pedimos es que lo hagas lo mejor que puedas. Tomara más o menos 15 minutos de tu tiempo para que respondes a la encuesta y que nos des tu muestra de heces.

**¿Cual son las buenas y malas cosas del estudio?**

Una de las buenas cosas del estudio es que te brinda la oportunidad de curarte de las lombrices. Eso quiere decir que te sentirás probablemente mejor y tendrás más atención en clase. También, otra buena cosa del estudio es que usaremos los resultados de este estudio para ayudar a los escolares de todo el mundo que tienen problemas con las lombrices.

Después de haber tomado la pastilla antiparasitaria, la gran mayoría de los niños no tendrá efectos secundarios. Sin embargo, hay una posibilidad que la pastilla te haga sentir un poco incomodo (dolor abdominal, diarrea, etc.). Sin embargo, eso no ocurre a menudo y la pequeña molestia dura poco tiempo. Si te sientes incomodo, comunica a tu madre, padre, o profesor y él te apoyara.

**¿Tendrás la obligación de contestar todas las preguntas y hacer todo lo que te pediremos?**

Las preguntas que haremos serán muy sencillas pero si no quieres contestar, infórmanos que no quieres contestar; no hay problema. Si te pedimos algo que no quieres hacer, infórmanos que no lo quieres hacer; no hay ningún problema – solamente tienes que informarnos. Nada te va a pasar si no quieres contestar las preguntas o no deseas hacer lo que te pedimos. No hay ninguna repuesta correcta o incorrecta a nuestras sencillas preguntas.

**¿Quién sabrá que participas en el estudio?**

Todas las cosas que dices y la información que escribamos será confidencial y guardada en un local con llave. Solamente los investigadores pueden ver tu información. Tu profesor, director, o padres no conocerán tus repuestas. Cuando hablaremos del estudio, nunca usaremos tu nombre o el de tus amigos.

**¿Tienes que participar en el estudio?**

No tienes que participar en el estudio si no lo deseas. Nadie se enojara si no quieres participar. Solamente necesitas informarnos que no quieres participar. También recuerda que si cambias de idea después acerca de tu participación, infórmanos que deseas participar o que ya no quieres participar.

**¿Tienes alguna pregunta?**

Puedes hacer preguntas en cualquier momento. Puedes hacerlas ahora o más tarde. Puedes hablar conmigo u otra persona en cualquier momento. También, puedes llamar a la coordinadora de investigación, Lic. Salomé Chapiama al 965 939153, línea gratuita 0800-1321 o presentarse a la oficina de la Asociación Civil Selva Amazónica, cual dirección es Urb. Jardín N 27, Fanning 4^ta^ Cuadra, Iquitos.

Si tienes alguna pregunta tus derechos como participante de una investigación, o alguna duda o inquietud sobre el tratamiento que recibirá en este estudio, comuníquese con el Dr. Andrés Paredes, Presidente Interino del Comité de Bioética de la Asociación Civil Impacta Salud y Educación al teléfono 01242-3072 anexo 130.

Declaración de asentimiento

He leído y comprendido los contenidos de este documento. He tenido la oportunidad de hacer preguntas y todas mis preguntas han sido resueltas a mi satisfacción. Mi participación es voluntaria. Entiendo que puedo retirarme del estudio a cualquier momento, sin consecuencias. Consiento mi participación en el estudio.

____________________ _________________________ ___________________

Nombre del participante Firma de participante Fecha (dd/mm/aaaa)

____________________ _________________________ ___________________

Nombre del entrevistador Firma de participante Fecha (dd/mm/aaaa)

____________________ _________________________ ___________________

Nombre del testigo Firma de testigo Fecha (dd/mm/aaaa)

| Informed Assent –  Child |
| --- |

**Annex 4:** Informed Assent Form – Students (English Version)

Effectiveness of a post-deworming education intervention to reduce infection and absenteeism in grade 5 school children of Belén


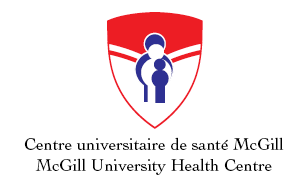

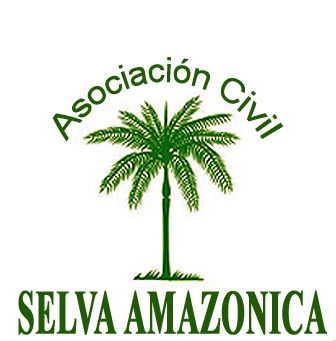

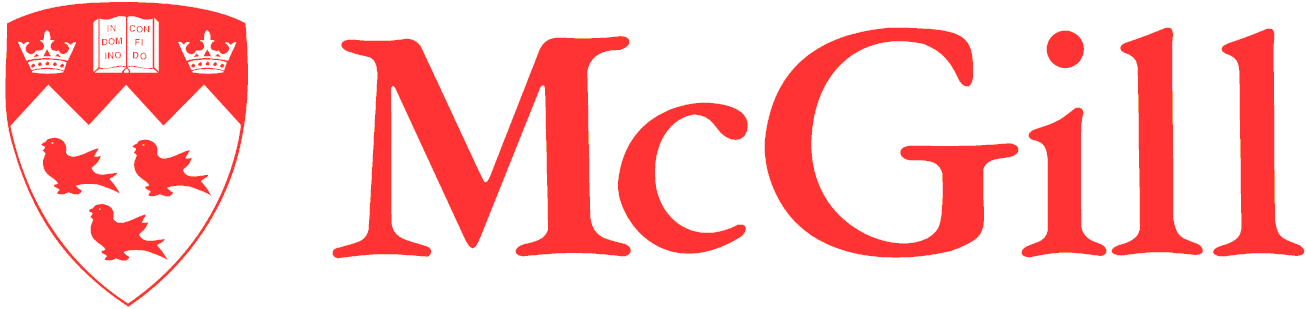


**Investigators and Institutions**

***Canada***: Dr. Theresa W. Gyorkos (Principal Investigator, Research Institute of the McGill University Health Centre - McGill University); Mathieu Maheu-Giroux (Coordinator, Research Institute of the McGill University Health Centre).

***Perú***: Dr. Martín Casapía (Project Director, Asociación Civil Selva Amazónica); Salomé Chapiama (Coordinator, Asociación Civil Selva Amazónica).

**Introduction**

Your parents have allowed me to talk to you about a project that I am working on with some colleagues. The project is on intestinal parasites that live inside your body and ways we can find to get rid of them. I am going to spend a few minutes telling you about our project, and then I am going to ask you if you are interested in taking part in the project.

**Why are we meeting with you?**

We want to tell you about a study that involves children like yourself in schools of Belén. We want to see if you would like to be in this study too. We want to find out ways to get rid of the intestinal parasites that can cause harm to your health.

**What will happen to you if you are in the study?**

If you decide to take part in this study there are some different things we will ask you about your health and habits. You will meet with one of our interviewers. We will measure your height and weight. We will also ask you to give us a small stool specimen. Then we will give you a pill, along with some fruit and fruit juice, that will get rid of the parasites. We will come back once or twice to ask you for another stool specimen to see if the parasites have come back or not. We will also ask you some other questions. While doing these things all you have to do is try your best. It will take you about 15 minutes to answer our questions and to give us your stool specimen.

**Are there good things and bad things about the study?**

One of the good things is that you will get rid of your parasites. This means that you will probably feel better and pay more attention in school. Another good thing is that we will use what we find out from this study to help school children around the world who have parasites.

After taking the parasite pill, most children will not have any reaction to it at all. But one bad thing could be that some children may feel a bit upset and have some stomach pain or diarrhea. The good thing is that this usually does not happen at all or it is over very fast. If you feel upset after taking the pill, you just need to tell your mother or father or teacher and they will take care of you.

**Will you have to answer all of the questions and do everything you are asked to do?**

The questions we ask you will be easy questions but if you don’t want to answer, then you don’t have to. Just tell us. Also, if you don’t want to do something else we ask you to do, it’s all right; just tell us that you don’t want to do it. Nothing bad will happen if you don’t want to tell us something or if you don’t want to do something that we have asked you to do. There is no right or wrong answer to any questions.

**Who will know that you are in the study?**

The things you tell us and any information that we have about you will be kept in a locked place. Only the researchers will be allowed to see it. Your teachers will not see it and your parents won’t see it. When we talk about this study, we will never use your name or any name.

**Do you have any questions?**

You can ask questions at any time. You can ask now or you can ask later. You can talk to me or you can talk to someone else at any time during the study.  You can call our Project Coordinator, Salomé Chapiama at 965-939153 (cellular), 221341 extension 151 (landline), or 0800-1321 (toll free). You can also present yourself at our office, at Urb. Jardín N 27, Fanning 4^ta^ Cuadra, Iquitos.

If you have any question about your rights as participants in this study or any concern regarding the deworming drug, you can call Dr. Andrés Paredes, ‘Presidente Interino del Comité de Bioética de la Asociación Civil Impacta Salud y Educación’ at 01242-3072 extension 130.

Declaration of Assent

I have read the contents of this document. I have had the opportunity to ask questions and all of them were answered satisfactorily. My participation in this study is free and voluntary. I know that I can withdraw from this study at any time if I want, without any adverse consequences. I agree to participate.

____________________ _________________________ ___________________

Name of Child Signature Date (dd/mm/yyyy)

____________________ _________________________ ___________________

Name of Interviewer Signature Date (dd/mm/yyyy)

____________________ _________________________ ___________________

Name of Witness Signature Date (dd/mm/yyyy)

**Annex 5:** Questionnaire (Spanish Version)

**Efectividad de una intervención educativa, post desparasitación, para reducir las lombrices y la inasistencia en los escolares de 5^to^ grado, Belén**

**Identificación de la escuela, niño(a), entrevistador**

| Institución educativa  _____________________  Nombre del profesor /clase  ______________________ | Nombre del niño/a  _________________________________  Código de identificación   \|  \| \| --- \| | entrevistador  _____________________  fecha de entrevista (dd/mm/aaaa)   \| **/ /** \| \| --- \| |
| --- | --- | --- | --- | --- |

**criterios de elegibilidad**

| **A** | ¿Tiene consentimiento de sus padres? | \| **⃞** \| Si \| *Realice la pregunta B* \| **⃞** \| No \| *Excluir su participación* \| \| --- \| --- \| --- \| --- \| --- \| --- \| |
| --- | --- | --- | --- | --- | --- | --- | --- | --- |
| **B** | ¿Tiene asentimiento del niño? | \| **⃞** \| Si \| *Realice la pregunta 1* \| **⃞** \| No \| *Excluir su participación* \| \| --- \| --- \| --- \| --- \| --- \| --- \| |

**Medidas tomada por el entrevistador**

| **1** | ¿Indique en qué turno está matriculado el niño? | \| **⃞** \| Mañana \| (1) \| **⃞** \| Tarde \| (2) \| **⃞** \| Noche \| (3) \| \| --- \| --- \| --- \| --- \| --- \| --- \| --- \| --- \| --- \| |
| --- | --- | --- | --- | --- | --- | --- | --- | --- | --- | --- | --- |
| **2** | ¿Cuál es el sexo del niño? | \| **⃞** \| Femenino \| (1) \|  \| **⃞** \| Masculino \| (0) \| \| --- \| --- \| --- \| --- \| --- \| --- \| --- \| |
| **3** | ¿Cuándo es tu cumpleaños? (dd/mm/aaaa) | \| **/ /** \| Edad \|  \| *No sabe* \| **⃞** \| \| --- \| --- \| --- \| --- \| --- \| |
| **4** | Peso del niño | \|  \|  \| Kg \|  \| *No se peso* \| **⃞** \| \| --- \| --- \| --- \| --- \| --- \| --- \| |
| **5** | Talla del niño | \|  \|  \| Talla \|  \| *No se tallo* \| **⃞** \| \| --- \| --- \| --- \| --- \| --- \| --- \| |
| **6** | ¿Tiene las uñas sucias o largas? | \| **⃞** \| Si \| (1) \| **⃞** \| No \| (0) \| **⃞** \| *No sabe* \| (99) \| \| --- \| --- \| --- \| --- \| --- \| --- \| --- \| --- \| --- \| |

**Nivel de conocimiento sobre los bichos**

| **i** | **A-** ¿Conoces cómo se transmiten los bichos (parásitos intestinales)? | | | | \| **⃞** \| Si \| (1) \|  \| **⃞** \| No \| (0) \| \| --- \| --- \| --- \| --- \| --- \| --- \| --- \| | |
| --- | --- | --- | --- | --- | --- | --- | --- | --- | --- | --- | --- | --- | --- |
|  | **B-** ¿Si, cómo?   \| **⃞** *No aplica (NA)* \| \| --- \| | \| 1) \|  \| \| --- \| --- \| \| 2) \|  \| \| 3) \|  \| \| 4) \|  \| | | | | |
| **II** | **A**- ¿Conoces por qué los bichos son dañinos para tu salud? | | | | \| **⃞** \| Si \| (1) \|  \| **⃞** \| No \| (0) \| \| --- \| --- \| --- \| --- \| --- \| --- \| --- \| | |
|  | **B-** ¿Si, Por qué son dañinos?   \| **⃞** *No aplica (NA)* \| \| --- \| | | | \| 1) \|  \| \| --- \| --- \| \| 2) \|  \| \| 3) \|  \| \| 4) \|  \| | | |
| **III** | **A**- ¿Conoces cómo puedes evitar los bichos? | | | | | \| **⃞** \| Si \| (1) \|  \| **⃞** \| No \| (0) \| \| --- \| --- \| --- \| --- \| --- \| --- \| --- \| |
|  | **B-** ¿Si, cómo?   \| **⃞** *No aplica (NA)* \| \| --- \| | | \| 1) \|  \| \| --- \| --- \| \| 2) \|  \| \| 3) \|  \| \| 4) \|  \| | | | |

**Encuesta sobre factores de riesgo**

| **7** | | **A-** ¿En qué comunidad /localidad vives?  **B-** ¿En qué calle, pasaje, manzana, sector o lote se ubica tu casa? | | \| Localidad: .................................................. \| **⃞** *No sabe* (99) \| \| --- \| --- \| \| Ubicación: .................................................  .................................................................... \| **⃞** *No sabe* (99)  **⃞** *No aplica* (NA) \| | |
| --- | --- | --- | --- | --- | --- | --- | --- | --- | --- |
| **8** | | ¿Tu casa está hecha de material noble o rústica? | | \| **⃞** \| Noble \| (1) \| **⃞** \| Rústica \| (0) \| **⃞** \| *No sabe* \| (99) \| \| --- \| --- \| --- \| --- \| --- \| --- \| --- \| --- \| --- \| | |
| **9** | | ¿En tu casa, cocinan con gas, kerosene, carbón, o leña? | | \| **⃞** \| Gas \| (0) \| **⃞** \| Carbón \| (2) \| **⃞** \| Otros_____ \| (4) \| \| --- \| --- \| --- \| --- \| --- \| --- \| --- \| --- \| --- \| \| **⃞** \| Kerosene \| (1) \| **⃞** \| Leña \| (3) \| **⃞** \| *No sabe* \| *(99)* \| | |
| **10** | | ¿Tienes energía eléctrica en casa? | | \| **⃞** \| Si \| (1) \| **⃞** \| No \| (0) \| **⃞** \| *No sabe* \| (99) \| \| --- \| --- \| --- \| --- \| --- \| --- \| --- \| --- \| --- \| | |
| **11** | | ¿Tienes radio en casa? | | \| **⃞** \| Si \| (1) \| **⃞** \| No \| (0) \| **⃞** \| *No sabe* \| (99) \| \| --- \| --- \| --- \| --- \| --- \| --- \| --- \| --- \| --- \| | |
| **12** | | ¿Tienes televisor en casa? | | \| **⃞** \| Si \| (1) \| **⃞** \| No \| (0) \| **⃞** \| *No sabe* \| (99) \| \| --- \| --- \| --- \| --- \| --- \| --- \| --- \| --- \| --- \| | |
| **13** | | **A-** ¿Tienes agua potable en tu casa (agua de grifo)? | | \| **⃞** \| Si \| (1) \| **⃞** \| No \| (0) \| **⃞** \| *No sabe* \| (99) \| \| --- \| --- \| --- \| --- \| --- \| --- \| --- \| --- \| --- \| | |
|  |  | **B-** ¿En caso que no, de dónde se abastecen de agua en tu casa?   \| **⃞** *No aplica (NA)* \| \| --- \| | | \| **⃞** \| Grifo vecino \| (0) \|  \| **⃞** \| Tanque \| (4) \| \| --- \| --- \| --- \| --- \| --- \| --- \| --- \| \| **⃞** \| Río \| (1) \|  \| **⃞** \| Pileta publica \| (5) \| \| **⃞** \| Pozo \| (2) \|  \| **⃞** \| Otros____________ \| (6) \| \| **⃞** \| Cisterna \| (3) \|  \| **⃞** \| *No sabe* \| (99) \| | |
| **14** | | ¿En casa tomas agua directo o tratada (hervida o clorada)? | | \| **⃞** \| Directo \| (1) \| **⃞** \| Tratada \| (0) \| **⃞** \| *No sabe* \| (99) \| \| --- \| --- \| --- \| --- \| --- \| --- \| --- \| --- \| --- \| | |
| **15** | | ¿Tu baño se ubica adentro o afuera de tu casa? | | \| **⃞** \| Adentro \| (1) \| **⃞** \| Afuera \| (0) \| **⃞** \| *No sabe* \| (99) \| \| --- \| --- \| --- \| --- \| --- \| --- \| --- \| --- \| --- \| | |
| **16** | | ¿Tu baño está conectado a tubo de desagüe, a caño, a río, o a pozo ciego? | | \| **⃞** \| Desagüe \| (0) \| **⃞** \| Río \| (2) \| \| --- \| --- \| --- \| --- \| --- \| --- \| \| **⃞** \| Caño \| (1) \| **⃞** \| Pozo ciego o silo \| (3) \| \| **⃞** *No sabe* (99) \| \| \| \| \| \| | |
| **17** | | ¿Te bañas en el río Itaya – siempre, a veces, o nunca? | | \| **⃞** \| Siempre \| (0) \| **⃞** \| Nunca \| (2) \| \| --- \| --- \| --- \| --- \| --- \| --- \| \| **⃞** \| A veces \| (1) \| **⃞** \| *No sabe* \| (99) \| | |
| **18** | | ¿Haces el dos en campo abierto (huerta o patio) - siempre, a veces, o nunca? | | \| **⃞** \| Siempre \| (0) \| **⃞** \| Nunca \| (2) \| \| --- \| --- \| --- \| --- \| --- \| --- \| \| **⃞** \| A veces \| (1) \| **⃞** \| *No sabe* \| (99) \| | |
| **19** | | ¿Te limpias con papel higiénico cuando haces el dos - siempre, a veces, o nunca? | | \| **⃞** \| Siempre \| (0) \| **⃞** \| Nunca \| (2) \| \| --- \| --- \| --- \| --- \| --- \| --- \| \| **⃞** \| A veces \| (1) \| **⃞** \| *No sabe* \| (99) \| | |
| **20** | | **A-** ¿Te lavas las manos después de ir al baño – siempre, a veces, o nunca?  **B-** ¿Cómo te lavas las manos después de ir al baño -con agua sola o jabón y agua?   \| **⃞** *No aplica (NA)* \| \| --- \|   **C-** ¿Si es con jabón, lo usas siempre, a veces, o nunca?   \| **⃞** *No aplica (NA)* \| \| --- \| | | \| **⃞** \| Siempre \| (0) \| **⃞** \| Nunca \| (2) \| \| --- \| --- \| --- \| --- \| --- \| --- \| \| **⃞** \| A veces \| (1) \| **⃞** \| *No sabe* \| (99) \|  \| **⃞** \| Agua \| (0) \| **⃞** \| Jabón y agua \| (1) \| **⃞** \| *No sabe* \| (99) \| \| --- \| --- \| --- \| --- \| --- \| --- \| --- \| --- \| --- \|  \| **⃞** \| Siempre \| (0) \| **⃞** \| Nunca \| (2) \| \| --- \| --- \| --- \| --- \| --- \| --- \| \| **⃞** \| A veces \| (1) \| **⃞** \| *No sabe* \| (99) \| | |
| **21** | | **A-** ¿Te lavas las manos antes de comer – siempre, a veces, o nunca?  **B-** Cómo te lavas las manos antes de comer -con agua sola o jabón y agua?   \| **⃞** *No aplica (NA)* \| \| --- \|   **C-** ¿Si es con jabón, lo usas siempre, a veces, o nunca?   \| **⃞** *No aplica (NA)* \| \| --- \| | | \| **⃞** \| Siempre \| (0) \| **⃞** \| Nunca \| (2) \| \| --- \| --- \| --- \| --- \| --- \| --- \| \| **⃞** \| A veces \| (1) \| **⃞** \| *No sabe* \| (99) \|  \| **⃞** \| Agua \| (0) \| **⃞** \| Jabón y agua \| (1) \| **⃞** \| *No sabe* \| (99) \| \| --- \| --- \| --- \| --- \| --- \| --- \| --- \| --- \| --- \|  \| **⃞** \| Siempre \| (0) \| **⃞** \| Nunca \| (2) \| \| --- \| --- \| --- \| --- \| --- \| --- \| \| **⃞** \| A veces \| (1) \| **⃞** \| *No sabe* \| (99) \| | |
| **22** | | **A-** ¿Cuál es tu fruta favorita?  **B-** ¿Lavas tus frutas antes de comer - siempre, a veces, o nunca? | | Fruta: ………………………………….   \| **⃞** \| Nunca \| (0) \| **⃞** \| Siempre \| (2) \| \| --- \| --- \| --- \| --- \| --- \| --- \| \| **⃞** \| A veces \| (1) \| **⃞** \| *No sabe* \| (99) \| | |
| **26** | | ¿Caminas descalzo(a) – siempre, a veces, o nunca? | | \| **⃞** \| Nunca \| (0) \| **⃞** \| Siempre \| (2) \| \| --- \| --- \| --- \| --- \| --- \| --- \| \| **⃞** \| A veces \| (1) \| **⃞** \| *No sabe* \| (99) \| | |
| **27** | | ¿Cuándo estás en casa, usas zapatos o sandalias? | | \| **⃞** \| No usa \| (0) \| **⃞** \| Zapato \| (2) \| \| --- \| --- \| --- \| --- \| --- \| --- \| \| **⃞** \| Sandalias \| (1) \| **⃞** \| *No sabe* \| (99) \| | |
| **28** | | ¿En qué actividades del día estas descalzo(a)?   \| **⃞** *No aplica (NA)* \| \| --- \| | | A- Actividad: ………..………………………………….  B- Actividad: ………..………………………………….  C- Actividad: ………..…………………………………. | |
| **29** | | **A-** ¿Tus padres o tu profesor te dieron purgante (te desparasitaron)?  **B-** ¿En caso que si, cuándo fue la última vez que te dieron purgante? | | \| **⃞** \| Si \| (1) \| **⃞** \| No \| (0) \| **⃞** \| *No sabe* \| (99) \| \| --- \| --- \| --- \| --- \| --- \| --- \| --- \| --- \| --- \|  \|  \| Meses \| **⃞** \| Más de un año (13) \| **⃞**  **⃞** \| *No sabe (99)*  No aplica (NA) \| \| --- \| --- \| --- \| --- \| --- \| --- \| | |
| **30** | | **A-** ¿Cuántas personas viven en tu casa? Descríbelos…  **B-** ¿Cuántos niños menores de 12 años viven en tu casa? | | \|  \| Personas  (incluyéndote) \| **⃞** \| *No sabe (99)* \| \| --- \| --- \| --- \| --- \| \|  \| Niños \| **⃞** \| *No sabe (99)* \| | |
|  | **Comentarios:** | |  | |  |
|  |  | | | |  |
|  |  | | | |  |

**Annex 6:** Questionnaire (English Version)

**Effectiveness of a post-deworming education intervention to reduce infection and absenteeism in grade 5 school children**

**School, Child, and Interviewer Identification**

| School name  _____________________  Name of Teacher/Class  ______________________ | Name of Child:  _________________________________  Unique Id number   \|  \| \| --- \| | Name of Interviewer  _____________________  Date of Interview (dd/mm/aaaa)   \| **/ /** \| \| --- \| |
| --- | --- | --- | --- | --- |

**Eligibility requirements**

| **A** | Has the parent consented? | \| **⃞** \| Yes \| *Go to question B* \| **⃞** \| No \| *Stop interview* \| \| --- \| --- \| --- \| --- \| --- \| --- \| |
| --- | --- | --- | --- | --- | --- | --- | --- | --- |
| **B** | Has the child assented? | \| **⃞** \| Yes \| *Go to question 1* \| **⃞** \| No \| *Stop interview* \| \| --- \| --- \| --- \| --- \| --- \| --- \| |

**Measures taken by the interviewer**

| **1** | Indicate if child attends morning, afternoon, or evening class | \| **⃞** \| Morning \| (1) \| **⃞** \| Afternoon \| (2) \| **⃞** \| Evening \| (3) \| \| --- \| --- \| --- \| --- \| --- \| --- \| --- \| --- \| --- \| |
| --- | --- | --- | --- | --- | --- | --- | --- | --- | --- | --- | --- |
| **2** | What is the sex of the child? | \| **⃞** \| Girl \| (1) \|  \| **⃞** \| Boy \| (0) \| \| --- \| --- \| --- \| --- \| --- \| --- \| --- \| |
| **3** | When is the child’s birthday? (dd/mm/aaaa) | \| **/ /** \| Age \|  \| *Don’t know* \| **⃞** \| \| --- \| --- \| --- \| --- \| --- \| |
| **4** | Weight of the child? | \|  \|  \| Kg \|  \| *(Not measured)* \| **⃞** \| \| --- \| --- \| --- \| --- \| --- \| --- \| |
| **5** | Height of the child? | \|  \|  \| Meters \|  \| *(Not measured)* \| **⃞** \| \| --- \| --- \| --- \| --- \| --- \| --- \| |
| **6** | Are the child’s fingernails dirty? | \| **⃞** \| Yes \| (1) \| **⃞** \| No \| (0) \| **⃞** \| *Don’t know* \| (99) \| \| --- \| --- \| --- \| --- \| --- \| --- \| --- \| --- \| --- \| |

**Level of Knowledge on Soil-transmitted helminths**

| **i** | **A-** Do you know how intestinal worms / parasites are transmitted? | | | | \| **⃞** \| Yes \| (1) \|  \| **⃞** \| No \| (0) \| \| --- \| --- \| --- \| --- \| --- \| --- \| --- \| | |
| --- | --- | --- | --- | --- | --- | --- | --- | --- | --- | --- | --- | --- | --- |
|  | **B-** If yes, how?   \| **⃞** *Not applicable (NA)* \| \| --- \| | \| 1) \|  \| \| --- \| --- \| \| 2) \|  \| \| 3) \|  \| \| 4) \|  \| | | | | |
| **II** | **A**- Do you know why worms / parasites are bad for your health? | | | | \| **⃞** \| Yes \| (1) \|  \| **⃞** \| No \| (0) \| \| --- \| --- \| --- \| --- \| --- \| --- \| --- \| | |
|  | **B-** If yes, why are they bad?   \| **⃞** *Not applicable (NA)* \| \| --- \| | | | \| 1) \|  \| \| --- \| --- \| \| 2) \|  \| \| 3) \|  \| \| 4) \|  \| | | |
| **III** | **A**- Do you know how you can avoid getting these worms / parasite infection? | | | | | \| **⃞** \| Yes \| (1) \|  \| **⃞** \| No \| (0) \| \| --- \| --- \| --- \| --- \| --- \| --- \| --- \| |
|  | **B-** If yes, how?   \| **⃞** *Not applicable (NA)* \| \| --- \| | | \| 1) \|  \| \| --- \| --- \| \| 2) \|  \| \| 3) \|  \| \| 4) \|  \| | | | |

**Questionnaire on risk factors**

| **7** | | **A-** Where do you live?  **B-** Where do you live (street, passage, sector, block, lot)? | | \| Community: ............................................... \| **⃞** *Don’t know* (99) \| \| --- \| --- \| \| Address: .................................................  .................................................................... \| **⃞** *Don’t know* (99)  **⃞** *Not applicable* (NA) \| | |
| --- | --- | --- | --- | --- | --- | --- | --- | --- | --- |
| **8** | | Is your house made out of noble or rustic materials? | | \| **⃞** \| Noble \| (1) \| **⃞** \| Rustic \| (0) \| **⃞** \| *Don’t know* \| (99) \| \| --- \| --- \| --- \| --- \| --- \| --- \| --- \| --- \| --- \| | |
| **9** | | In your house, do you Cook with gas, kerosene, coal, o wood? | | \| **⃞** \| Gas \| (0) \| **⃞** \| Coal \| (2) \| **⃞** \| Others____ \| (4) \| \| --- \| --- \| --- \| --- \| --- \| --- \| --- \| --- \| --- \| \| **⃞** \| Kerosene \| (1) \| **⃞** \| Wood \| (3) \| **⃞** \| *Don’t know* \| *(99)* \| | |
| **10** | | Do you have electricity at your house? | | \| **⃞** \| Yes \| (1) \| **⃞** \| No \| (0) \| **⃞** \| *Don’t know* \| (99) \| \| --- \| --- \| --- \| --- \| --- \| --- \| --- \| --- \| --- \| | |
| **11** | | Does your family own a radio? | | \| **⃞** \| Yes \| (1) \| **⃞** \| No \| (0) \| **⃞** \| *Don’t know* \| (99) \| \| --- \| --- \| --- \| --- \| --- \| --- \| --- \| --- \| --- \| | |
| **12** | | Does your family own a television? | | \| **⃞** \| Yes \| (1) \| **⃞** \| No \| (0) \| **⃞** \| *Don’t know* \| (99) \| \| --- \| --- \| --- \| --- \| --- \| --- \| --- \| --- \| --- \| | |
| **13** | | **A-** Do you have potable water in your house? | | \| **⃞** \| Yes \| (1) \| **⃞** \| No \| (0) \| **⃞** \| *Don’t know* \| (99) \| \| --- \| --- \| --- \| --- \| --- \| --- \| --- \| --- \| --- \| | |
|  |  | **B-** If not, where do you get your water from?   \| **⃞** *Not applicable (NA)* \| \| --- \| | | \| **⃞** \| Neighbour \| (0) \|  \| **⃞** \| Tank \| (4) \| \| --- \| --- \| --- \| --- \| --- \| --- \| --- \| \| **⃞** \| River \| (1) \|  \| **⃞** \| Public fountain \| (5) \| \| **⃞** \| Well \| (2) \|  \| **⃞** \| Others___________ \| (6) \| \| **⃞** \| Truck \| (3) \|  \| **⃞** \| *Don’t know* \| (99) \| | |
| **14** | | In your house do you drink your water directly or do you treat it (boiling or bleaching)? | | \| **⃞** \| Directly \| (1) \| **⃞** \| Treated \| (0) \| **⃞** \| *Don’t know* \| (99) \| \| --- \| --- \| --- \| --- \| --- \| --- \| --- \| --- \| --- \| | |
| **15** | | At home, is your latrine inside or outside the house? | | \| **⃞** \| Inside \| (1) \| **⃞** \| Outside \| (0) \| **⃞** \| *Don’t know* \| (99) \| \| --- \| --- \| --- \| --- \| --- \| --- \| --- \| --- \| --- \| | |
| **16** | | Is your latrine connected with the sewage system, a ditch, the river, or a well? | | \| **⃞** \| Sewage \| (0) \| **⃞** \| River \| (2) \| \| --- \| --- \| --- \| --- \| --- \| --- \| \| **⃞** \| Ditch \| (1) \| **⃞** \| Well \| (3) \| \| **⃞** *Don’t know* (99) \| \| \| \| \| \| | |
| **17** | | Do you bath in the Itaya river – always, sometimes, or never? | | \| **⃞** \| Always \| (0) \| **⃞** \| Never \| (2) \| \| --- \| --- \| --- \| --- \| --- \| --- \| \| **⃞** \| Sometimes \| (1) \| **⃞** \| *Don’t know* \| (99) \| | |
| **18** | | Do you defecate (#2) in the open air – always, sometimes, or never? | | \| **⃞** \| Always \| (0) \| **⃞** \| Never \| (2) \| \| --- \| --- \| --- \| --- \| --- \| --- \| \| **⃞** \| Sometimes \| (1) \| **⃞** \| *Don’t know* \| (99) \| | |
| **19** | | Do you use toilet paper to wipe your bum after you have defecated (#2) – always, sometimes, or never? | | \| **⃞** \| Always \| (0) \| **⃞** \| Never \| (2) \| \| --- \| --- \| --- \| --- \| --- \| --- \| \| **⃞** \| Sometimes \| (1) \| **⃞** \| *Don’t know* \| (99) \| | |
| **20** | | **A-** Do you wash your hands after going to the bathroom – always, sometimes, or never?  **B-** How do you wash your hands after going to the bathroom – with water only or with soap and water?   \| **⃞** *Not applicable (NA)* \| \| --- \|   **C-** If with soap and water, do you use soap always, sometimes, or never?   \| **⃞** *Not applicable (NA)* \| \| --- \| | | \| **⃞** \| Always \| (0) \| **⃞** \| Never \| (2) \| \| --- \| --- \| --- \| --- \| --- \| --- \| \| **⃞** \| Sometimes \| (1) \| **⃞** \| *Don’t know* \| (99) \|  \| **⃞** \| Water \| (0) \| **⃞** \| Soap and water \| (1) \| **⃞** \| *Don’t know* \| (99) \| \| --- \| --- \| --- \| --- \| --- \| --- \| --- \| --- \| --- \|  \| **⃞** \| Always \| (0) \| **⃞** \| Never \| (2) \| \| --- \| --- \| --- \| --- \| --- \| --- \| \| **⃞** \| Sometimes \| (1) \| **⃞** \| *Don’t know* \| (99) \| | |
| **21** | | **A-** Do you wash your hands before eating – always, sometimes, or never?  **B-** How do you wash your hands before eating – with water only or with soap and water?   \| **⃞** *Not applicable (NA)* \| \| --- \|   **C-** ¿ If with soap and water, do you use soap always, sometimes, or never?   \| **⃞***Not applicable (NA)* \| \| --- \| | | \| **⃞** \| Always \| (0) \| **⃞** \| Never \| (2) \| \| --- \| --- \| --- \| --- \| --- \| --- \| \| **⃞** \| Sometimes \| (1) \| **⃞** \| *Don’t know* \| (99) \|  \| **⃞** \| Water \| (0) \| **⃞** \| Soap and water \| (1) \| **⃞** \| *Don’t know* \| (99) \| \| --- \| --- \| --- \| --- \| --- \| --- \| --- \| --- \| --- \|  \| **⃞** \| Always \| (0) \| **⃞** \| Never \| (2) \| \| --- \| --- \| --- \| --- \| --- \| --- \| \| **⃞** \| Sometimes \| (1) \| **⃞** \| *Don’t know* \| (99) \| | |
| **22** | | **A-** What is your favourite fruit that you eat?  **B-** Do you was your fruits before eating - always, sometimes, or never? | | Fruit: ………………………………….   \| **⃞** \| Never \| (0) \| **⃞** \| Always \| (2) \| \| --- \| --- \| --- \| --- \| --- \| --- \| \| **⃞** \| Sometimes \| (1) \| **⃞** \| *Don’t know* \| (99) \| | |
| **26** | | Do you walk barefoot - always, sometimes, or never? | | \| **⃞** \| Never \| (0) \| **⃞** \| Always \| (2) \| \| --- \| --- \| --- \| --- \| --- \| --- \| \| **⃞** \| Sometimes \| (1) \| **⃞** \| *Don’t know* \| (99) \| | |
| **27** | | When you are at home do you prefer to use sandals or shoes? | | \| **⃞** \| Does not use any \| (0) \| **⃞** \| Shoes \| (2) \| \| --- \| --- \| --- \| --- \| --- \| --- \| \| **⃞** \| Sandals \| (1) \| **⃞** \| *Don’t know* \| (99) \| | |
| **28** | | In which activities of the day are you barefoot?   \| **⃞** *Not applicable (NA)* \| \| --- \| | | A- Activity: ………..………………………………….  B- Activity: ………..………………………………….  C- Activity: ………..…………………………………. | |
| **29** | | **A-** Did your parents or your professor gave you a deworming pill?  **B-** If yes, when was the last time they gave you such deworming pill? | | \| **⃞** \| Yes \| (1) \| **⃞** \| No \| (0) \| **⃞** \| *Don’t know* \| (99) \| \| --- \| --- \| --- \| --- \| --- \| --- \| --- \| --- \| --- \|  \|  \| Months \| **⃞** \| More than a year (13) \| **⃞**  **⃞** \| *Don’t know (99)*  Not applicable (NA) \| \| --- \| --- \| --- \| --- \| --- \| --- \| | |
| **30** | | **A-** How many people live in your house? Enumerate them…  **B-** How many children younger than 12 years old live in your house? | | \|  \| People  (including you) \| **⃞** \| *Don’t know (99)* \| \| --- \| --- \| --- \| --- \| \|  \| Children \| **⃞** \| *Don’t know (99)* \| | |
|  | **Comments:** | |  | |  |
|  |  | | | |  |
|  |  | | | |  |
